# Applicability of Different Hydraulic Parameters to Describe Soil Detachment in Eroding Rills

**DOI:** 10.1371/journal.pone.0064861

**Published:** 2013-05-24

**Authors:** Stefan Wirtz, Manuel Seeger, Andreas Zell, Christian Wagner, Jean-Frank Wagner, Johannes B. Ries

**Affiliations:** 1 Department of Physical Geography, Trier University, Trier, Germany; 2 Department of Land Degradation and Development, Wageningen University, Wageningen, The Netherlands; 3 Department 7.3- Technical Physics, Saarland University, Saarbrücken, Germany; 4 Department of Geology, Trier University, Trier, Germany; Plymouth University, United Kingdom

## Abstract

This study presents the comparison of experimental results with assumptions used in numerical models. The aim of the field experiments is to test the linear relationship between different hydraulic parameters and soil detachment. For example correlations between shear stress, unit length shear force, stream power, unit stream power and effective stream power and the detachment rate does not reveal a single parameter which consistently displays the best correlation. More importantly, the best fit does not only vary from one experiment to another, but even between distinct measurement points. Different processes in rill erosion are responsible for the changing correlations. However, not all these procedures are considered in soil erosion models. Hence, hydraulic parameters alone are not sufficient to predict detachment rates. They predict the fluvial incising in the rill's bottom, but the main sediment sources are not considered sufficiently in its equations. The results of this study show that there is still a lack of understanding of the physical processes underlying soil erosion. Exerted forces, soil stability and its expression, the abstraction of the detachment and transport processes in shallow flowing water remain still subject of unclear description and dependence.

## Introduction

Soil erosion models use different composite factors to describe and predict soil detachment and transport capacity. The most frequently used factors are average shear stress [1]–[4], unit length shear force [5], stream power [4], [6]–[9], unit stream power [10], [11] and effective stream power [12], [13].

In most cases, a linear equation describes the relation between the hydraulic parameters mentioned above and the detachment rate. By exceeding a certain threshold, erosion by concentrated flow begins and detachment rate increases. This threshold has a positive x-axis intercept, which means that there is no detachment below this point.

Another option is to consider concentrated flow erosion as a nonlinear threshold phenomenon or as a two-part linear threshold phenomenon: below the threshold soil detachment takes place (first linear relationship) but after exceeding the threshold, detachment rate increases much faster (second linear relationship) [14]. But it is unclear if this linear relationship is really suitable.

Knapen et al. [14] calculated the correlation between shear stress, unit length shear force, stream power and Reynolds number and the detachment rate from several WEPP datasets. The best average correlation was determined for stream power with R^2^ = 0.59. The WEPP-used shear stress is a variable that reaches only low R^2^ values for all of the tested data sets. Knapen et al. [14] describes the shear stress as follows (p. 80 f.): “Although the use of flow shear stress as soil detachment predictor can be contested, critical shear stress (τ_cr_) and concentrated flow erodibility KC (…) have been selected as the most universal parameters to describe soil erosion resistance to concentrated flow.” The correlations between these factors and the soil detachment rate show very varying results. There is not a single parameter that always reveals the best correlation. These considerations lead to two main questions:

1. Are soil erosion, detachment and transport, directly dependent on water flow characteristics?
2. Are these concepts, as implemented in soil erosion models, suitable to describe rill erosion?

These questions have been tackled by many research groups that have been searching for the equation that suits their observations best [1]–[13], [15]–[42]. However, taking into consideration the numerous and variable results, a deeper insight into the rill erosion processes on hillslopes is essential. To get this insight, different strategies can be applied [43]: (1) Modelling, (2) laboratory experiments (3) field observations and (4) field experiments. Each of these methods shows different advantages and disadvantages.

Due to difficulties to measure certain parameters, models have to be calibrated. During this process, the phenomenon of equifinality can appear: different parameter sets show the same result. Another weakness of rill erosion models is that the model parameters are often adapted from river hydrodynamics equations. Govers and his colleagues [13], [44] showed that these equations are not suitable for rill erosion processes. Therefore, there is often a mismatch between model results and observed or measured “reality” [43]. Additionally, models only project the concepts of the designer, not necessarily the reality.

In laboratory experiments, the initial and boundary conditions are well controlled. Soil parameters are well known and rill forms and slope can be adapted to the specific question. Thus, physical laws can be tested in a well-defined environment. However, Giménez and Govers [5] showed that parameters determined under laboratory conditions are not easily transformable to natural environments. One disadvantage of former laboratory experiments or field observations is the fact that in most cases only total runoff and sediment output are measured while the relative contribution of the individual processes is not considered [45].

Field data currently reflect the reality as close as possible. Nevertheless, observations as well as experiments show certain disadvantages: (1) Measurement techniques may disturb the observed processes, (2) time scale of human observations is shorter than that of the process under study, (3) some processes cannot be measured directly or indirectly and (4) some processes are chaotic and the spatial and temporal variations are difficult to specify [43].

The relationship between soil detachment and hydraulic parameters used in soil erosion models is in most cases deduced from laboratory experiments but the transferability of these results to natural rills is not generally given. Our setup in natural rills enables to measure the input parameters for calculating hydraulic parameters combining the advantages of laboratory experiments with the advantages of testing natural rills.

The main purpose of the field experiments was to quantify in a detailed temporal and spatial resolution the soil erosion dynamics in natural rills under concentrated flow for comparison of the measured sediment dynamics with those calculated by means of the most common detachment and transport equations.

Specifically, this study's objectives are:

1. elucidating the relationship between hydraulic parameters such as shear stress, unit length shear force, unit stream power, stream power, effective stream power and the Reynolds number and soil detachment in natural rills,
2. providing an explanation why physically-based soil erosion models do not capture rill erosion processes and
3. addressing the question whether current modelling approaches are generally suited to describe rill erosion processes.

The overall aim of this study is to have a critical view on concepts for modelling rill erosion based on experiments performed in naturally developed rills.

## Materials and Methods

### Ethics Statement

No specific permits were required for the described field studies. The mayors of the towns next to the study sites or the owners of the fields were informed about the intended activities and were asked for permission. The test sites Freila, Negratin and Salada are abandoned fields which are sporadically used as pasture for goats or sheep and in Belerda the experiment was accomplished on an almond field. The locations Freila, Negratin end Salada are not privately-owned and permission was granted from the owner of the study site Belerda. None of the study sites are protected in any way and the field studies did not involve endangered or protected species.

### Study areas

The four study areas in Andalusia are located at Negratin, Freila, Salada and Belerda. UTM coordinates of the tested rills are given in Table 1.

**Table 1:** Description table of the experiments: Temperature and precipitation with the nearest meteorological station (INM).

Experiment	Meteorological station	Average annual temperature	Annual precipitation	Northing of the rill	Easting of the rill
Freila 1+3	Baza	14.2°C	368 mm	4154368	509860
Freila 2	Baza	14.2°C	368 mm	4154398	509826
Negratin	Baza	14.2°C	368 mm	4156324	505710
Salada	Embalse Valdeinfierno	13.4°C	311 mm	4187266	595761
Belerda	Granada	15.6°C	473 mm	4133440	478070

UTM 30 coordinates of the five tested rills are presented.

Freila 1 and Freila 3 are two experiments in the same rill.

#### Negratin and Freila

The areas are located within the Hoya de Baza sedimentary basin and composed of marls, in which calcareous Regosols have developed. The climate is semi-arid and vegetation is dominated by low shrubs and *Stipa tenacissima* grass tussocks. The land cover at the south side of the Negratin-dam is dominated by abandoned cereal fields, which are extensively grazed by sheep and agricultural land comprised mainly of cereal dry-farming and almond grooves [46].

#### Salada

Located at the SE-margin of the Betic range (SE-Spain), inside the penibetic complex. The area is composed of conglomerates with a clayey to loamy matrix, in which Regosols as well as to fairly developed (Calcic) Cambisols have developed. Vegetation is similar to that found in the Freila and Negratin-area. The climate is semi-arid too, but less accentuated than in the previously mentioned area [46]. Here the land use consists of rain fed agricultural areas (where cereals, olives, and almonds are cultivated), and abandoned or uncultivated areas.

#### Belerda

This test area is located in the Guadix basin. The parent material consists of tertiary and quaternary conglomerates, sands, silts and clays. The soil texture class following the FAO [47] is a silty clay loam. The land use is separated into cultivated areas, with almond and olive groves, and abandoned agricultural fields [48]. The climate is, though still semi-arid, characterised by higher average annual temperatures and precipitations in comparison with the other test zones.

The climatic parameters of the test fields are summarized in Table 1.

### Tested rills

**Figure 1:**
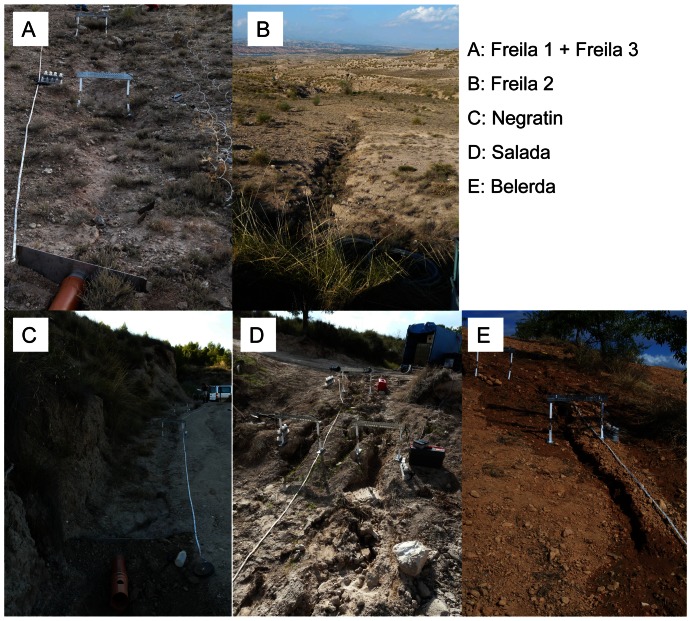
Photographies of the tested rills. Informations about the rills are presented in Table 2.

The main descriptors of the rills are summarized in Table 2. In this table, grain size class limits are from [49], texture class is determined following [47]. Photographies of the rills are presented in Figure 1.

**Table 2:** Rill parameters: Grain size class limits are from [49], texture class is determined following [47].

		Freila 1	Freila 2	Freila 3	Negratin	Salada	Belerda
	Ø Slope [°]	9.4	7.7	9.4	5.6	25.6	16.9
	Max. Slope [°]	15.2	14.1	15.2	12.9	7.3	12.5
	Tested flow length [m]	16	21	16	30	17	23
	Texture class	SL	SL	SL	SiL	SiCL	L
Gravel	>2000 µm [%]	30	30	30	1	1	13
Sand	2000-630 µm [%]	14	14	14	1	2	10
	630-200 µm [%]	14	14	14	5	2	10
	200-125 µm [%]	13	13	13	6	1	8
	125-63 µm [%]	16	16	16	11	7	17
Silt	63-20 µm [%]	13	13	13	11	17	13
	20-6.3 µm [%]	10	10	10	20	17	13
	6.3-2 µm [%]	11	11	11	24	24	14
Clay	<2 µm [%]	9	9	9	21	29	15
	Starting soilmoisture [% w/w]	3.1	3.5	3.1	3.1	5.8	2.4
	K_t_ [s^2^ m^0.5^ kg^−0.5^]	0.0090	0.0090	0.0090	0.0095	0.0096	0.0093
	Location WEPP dataset	Academy	Academy	Academy	Frederick	Mexico	Caribou
	Maximum width [m]	∼0.4	∼2.2	∼0.4	∼0.4	∼0.5	∼0.3
	Maximum depth [m]	∼0.05	∼0.7	∼0.05	∼0.2	∼0.25	∼0.15
	Vegetation cover [%]	∼40	∼40	∼40	∼0	∼15	∼5
	Rock fragment cover [%]	∼80	∼80	∼80	∼5	∼20	∼50
	Grain density [g cm^−3^]	2.69	2.69	2.69	2.65	2.66	2.61
	Dry bulk density [g cm^−3^]	1.44	1.55	1.44	1.57	1.52	1.68
	Org. material [%]	1.29	1.29	1.29	1.75	2.97	1.34
	Critical shear stress [Pa]	1.97	2.07	1.97	2.93	3.20	2.77
	Land use	rangeland	rangeland	rangeland	rangeland	rangeland	cropland

K_t_ is a transport coefficient, which has been adopted from the WEPP dataset. The WEPP-location is given. Measured values are starting soil moisture, maximum width, maximum depth, grain density, dry bulk density, org. material; parameters estimated in the field are vegetation cover and rock fragment cover; critical shear stress is calculated following WEPP.

The tested rills in Freila have developed on a sandy loam with high gravel content. Sand content is 57% with a relatively homogeneous contribution between coarse, medium, fine and very fine sand. The same is true in the silt fraction, the 34% are homogeneously contributed in the complete silt fraction between 63 and 2 µm. The rills show all a dense rock fragment cover and the highest vegetation cover of the four test sides.

In Negratin, the soil material is nearly gravel free, coarse, medium and fine sand also show low amounts, most of the fine material is in the grain size class <20 µm. The rock fragment cover in the rill is higher than the gravel content of the soil material thus it is possible that residual rock fragment accumulation has occurred.

In Salada the grain size distribution is similar to Negratin. The highest account of the fine soil material is in the class <63 µm. The residual rock fragment accumulation is formed even more clearly as in Negratin; the vegetation cover is relatively high compared to the other test sites.

The rill in Salada is the only rill that has developed in a field being used for agriculture. The soil material is composed by a mixture of all particle size classes from gravel to clay. The rock fragment cover is high compared to the other test sites and the vegetation cover comparatively low. This test site shows the highest dry bulk density which can be declared by the actual agricultural use.

### Rill experiment (RE)

The rill experiments consist of two runs: first the rill is tested under field conditions (run a); in a second run (run b), approximately 15 minutes later, the same rill is tested under almost saturated soil conditions. A constant discharge of 250 L (or 330 L, respectively) is maintained during 4 minutes (or 3 minutes, respectively), using a motor-driven pump, resulting in a total water inflow of 1000 L. Mobilisation of material at the inflow has been avoided.

The flow velocity within the rill is characterized by the travel time of the waterfront and of two colour tracers (started at 1 and 2 minutes of the experiment), measured for every meter using a chronograph. By means of this procedure, three velocity curves are recorded and changes in flow dynamics can be detected. As colour tracers, food colourings (E 124 (red) and E 13 (blue)) are used for reasons of safety.

The rill's slope is characterized by measuring with a spring bow of 1 m range and a digital spirit level. It must be considered that slope measuring provides only average slopes for 1 meter. A step or a knick-point in the rill is not accounted, but its position and height are recorded.

Four water samples are taken at three different measuring points (MP1–MP3). The first sample is taken as soon as the waterfront has reaching the sampling point, the second 30 seconds later, the third 90 second later, and the fourth 150 seconds later.

The (suspended) sediment concentration SSC is determined by filtration of the samples in laboratory [50].

At each measuring point, rill cross section is measured. With a laser rangefinder, the distance between sensor and rill bottom is measured in 0.002 m steps. This allows an accurate calculation of the rills cross section area and an estimation of the rills volume.

Water level is continuously measured by ultrasonic sensors at each measuring point.

### Descriptors for soil detachment

Soil detachment can be described by shear stress τ, unit length shear force Γ, stream power ω, unit stream power ω_U_ and effective stream power ω_eff_.
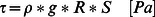
(1)

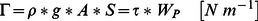
(2)

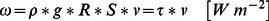
(3)

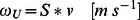
(4)

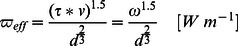
(5)with ρ = liquid density [kg m^−3^], g the gravitational acceleration (9.81 m s^−2^), R the hydraulic radius [m], A the flow cross section area [m^2^], *S* the effective slope (sin(slope angle)), W_P_ the wetted perimeter [m], v the flow velocity [m s^−1^] and d the water depth [m]; abbreviations of the units are Pa = Pascal, N = Newton, W = Watt.

Reynolds number describes the balance between the inertial flow forces represented by the product in the numerator and the viscous forces as described by the dynamic viscosity in the denominator. It is a criterion for stability of a flowing medium. When Reynolds number is small, viscous forces dominate the motion and inertial ones can be ignored whereas at high Reynolds numbers inertial forces dominate and it is often possible to ignore viscosity [51]. Reynolds Number Re is calculated as follows:
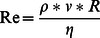
(6)with ρ = liquid density [ kg m^−3^], v = flow velocity [m s^−1^], R = hydraulic radius [m] and η = dynamic viscosity [Pa s].

Liquid density is calculated using sediment concentration and grain density. The use of water's density is not practicable due to sediment concentrations of more than 400 g L^−1^. Grain density was measured by a capillary pycnometer following DIN 18124 [52]. Flow velocity for each sample is interpolated between three measured velocities (arrival of the waterfront and arrival of the two colour tracers). Hydraulic radius and wetted cross section area can be calculated by measuring water level and the rill profile.

The viscosity of the sediment suspensions was measured with a shear rate controlled rheometer (Haake MARS from Thermo Fisher Scientific, Karlsruhe, Germany) and a cone-plate geometry with an angle of 2° and a diameter of 60 mm [53]. The shear rate γ is defined as:
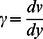
(7)with v = fluid velocity and y = the gap between the cone and base plate. The rheomter controls the shear rate and measures the shear stress τ, from which the viscosity η is calculated via
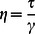
(8)The sample volume is always 2.0 ml and the cell is tempered to 20°C+/−0.01°C. Data points are taken at shear rates between 150 s^−1^ and 1500 s^−1^. The viscosity does not depend on the shear rate. This is according to theoretical considerations. For a suspension of monodisperse particles one expects a linear relation [54], [55] for volume concentrations up to approximately 10%.

Detachment rate D_R_ [kg s^−1^ m^−2^] is calculated from the measured sediment concentrations and different hydraulic parameters:
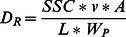
(9)with SSC = sediment concentration [g L^−1^ = kg m^−3^] and L = flow length [m].

For the calculation of the critical shear stress, the equations from the WEPP model [34] is used. The authors separate between “cropland with sand content >30%” and “rangeland”.
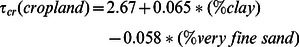
(10)

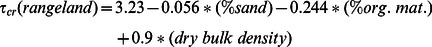
(11)For quantification of the different processes in the rill, the transport rate T_R_ [kg s^−1^] and the transport capacity T_C_ [kg s^−1^] are calculated:
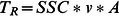
(12)

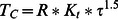
(13)Kt [s^2^ m^0.5^ kg^−0.5^] is a transport coefficient depending on soil substrate. The Kt value of the WEPP substrate which was most similar to the given test site conditions is used.

### Quantification of different erosion processes

Following shear stress based model concepts, the transport rate cannot exceed the transport capacity [56]. Shear stress of the flowing water controls also the detachment. Therefore the transport rate up to the transport capacity is considered here as shear stress dependent uptake. The transport rate exceeding the transport capacity is considered as shear stress independent erosion caused by processes such as bank failure and headcut retreat. The resulting quantities are set into relation and given in percent of total transport rate.

## Results

### Initial data

The used parameters show a wide range of data. In most cases (12 of 19), the standard deviation is higher than the mean values, the highest standard deviation – mean - percentage reaches the transport capacity (224%), the effective stream power (188.9%), the sediment concentration (168.3%) and detachment and transport rate (both 150%). The lowest percentage is calculated for sample density (0.5%). All initial data are presented in supporting information Tables S1, S2, S3, S4, S5, S6, S7, S8, S9, S10, S11, S12, S13, S14, S15, S16, S17, S18 and the statistical values of the data in Table 3.

**Table 3:** Descriptive statistics of the initial data.

variable	Maximum	Minimum	Mean	Standard Deviation	Percentage from Mean
SSC [g L^−1^]	422.30	0.001	52.15	87.78	168.3
D_R_ [kg s^−1^ m^−2^]	0.96	0.001	0.10	0.15	150.0
T_R_[kg s^−1^]	2.06	0.001	0.16	0.24	150.0
p [g cm^−3^]	1.26	1.00	1.03	0.005	0.5
Slope [°]	24.50	1.70	9.73	6.90	70.9
T_C_ [kg s^−1^]	3.38	0.001	0.25	0.56	224.0
v [m s^−1^]	2.94	0.04	0.79	0.49	62.0
η [kg s^−1^ m^−1^]	0.00311	0.00100	0.00126	0.00044	34.9
Water depth [cm]	21.00	0.20	3.99	4.23	106.0
A [cm^2^]	877.69	0.80	149.21	195.84	131.3
W_P_ [cm]	107.58	4.85	38.21	24.16	63.2
R [cm]	9.65	0.10	2.92	2.12	72.6
τ [Pa]	246.70	0.96	52.38	55.18	105.3
Г [N m^−1^]	172.58	0.10	23.99	35.10	146.3
ω [W m^−2^]	365.28	0.31	41.54	55.91	134.6
ω_U_ [m s^−1^]	0.88	0.001	0.14	0.17	121.4
ω_eff_ [W m^−1^]	37864.55	5.81	3807.14	7192.32	188.9
Re [ ]	86918.88	237.00	19053.94	16226.56	85.2
τ - τ_cr_ [Pa]	244.73	−1.46	49.89	55.11	110.5

SSC = sediment concentration, D_R_ = detachment rate, T_R_ = transport rate, p = sample density, T_C_ = transport capacity, v = flow velocity, η = dynamic viscosity, A = flow cross section, W_P_ = wetted perimeter, R = hydraulic radius, τ = shear stress, Г = unit length shear force, ω = stream power, ω_U_ = unit stream power, ω_eff_ = effective stream power, Re = Reynolds-Number, τ_cr_ = critical shear stress.

### Dynamic viscosity

**Figure 2:**
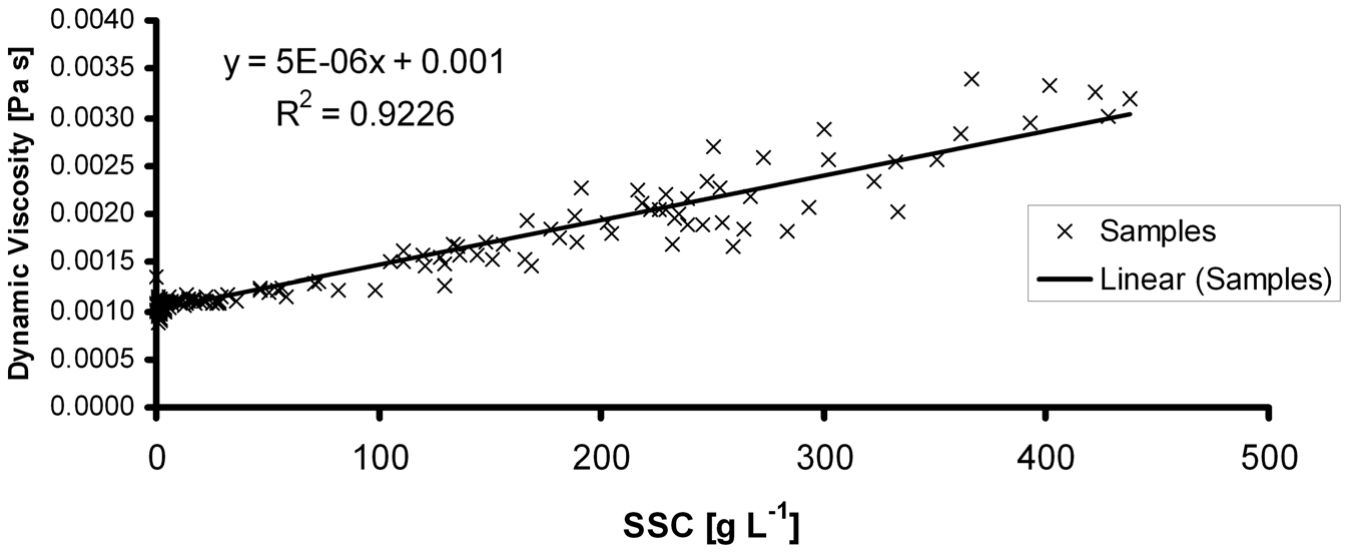
Correlation between sediment concentration of each sample and the measured dynamic viscosity. The linear correlation function and the R^2^ value is presented.

The dynamic viscosity of the liquid shows a clear positive correlation with sediment concentration, i.e. dynamic viscosity increases with sediment concentration (see Figure 2). However, clear deviations from the trend line were observed for samples with low sediment concentrations, which were often rich in transported organic material. The small branchlets with low weight imply a low sediment concentration, but in rheometer measurements, they tilt and a high shear stress is erroneously measured. The trend line equation has been calculated for samples from different test sites, the R^2^-value of 0.92 indicates that this equation can be used for further experiments.

### Correlations between detachment rate and hydraulic parameters

The R^2^ values of the correlations between the detachment rate and different hydraulic parameters show the complete possible range from R^2^ = 0 up to R^2^ = 0.99 (see Table S19). Trend lines are increasing, decreasing and almost constant and thus it is not possible to find any clear dependency. Notably, only 40 of 252 correlations (about 16%) show an increasing trend line with an R^2^ value≥0.7. Table 4 shows that the highest average R^2^-value is calculated for the (τ-τ_cr_) – detachment rate - relationship if all R^2^ values are used (0.53), if only the R^2^- values with increasing trend line are considered in calculation, the τ – detachment rate relationship shows the highest average R^2^ (0.55). Separating the experiments into two groups, Freila 1–3 with low sediment concentrations (LSSC) and Negratin, Salada, Belerda with high sediment concentrations (HSSC), the highest R^2^-values of the LSSC-experiments reach τ, Г and the (τ-τ_cr_) – detachment rate - relationship (0.65) if all values are used respectively the (τ-τ_cr_) – detachment rate - relationship (0.39) if only the R^2^ values≥0.7 with increasing trend lines are used. In the HSSC-experiments, the Г reaches the highest value (0.70) if all values are included and ω_eff_ (0.52) if only the R^2^ values≥0.7 with increasing trend lines are used, respectively.

**Table 4:** R^2^ - correlation values between different hydraulic parameters and the detachment rate.

	τ	Г	ω	ω_U_	ω_eff_	Re	τ - τ_cr_
all values	0.52	0.50	0.37	0.43	0.40	0.39	0.53
only values with increasing trendline	0.55	0.52	0.40	0.46	0.45	0.39	0.53
all Freila experiments	0.65	0.65	0.50	0.53	0.48	0.53	0.65
Negratin, Salada, Belerda all values	0.69	0.70	0.43	0.56	0.56	0.49	0.64
Freila only values with increasing trend line	0.38	0.36	0.25	0.33	0.32	0.26	0.39
Negratin, Salada, Belerda only values with increasing trend lines	0.44	0.41	0.39	0.43	0.52	0.35	0.45

τ = shear stress, Г = unit length shear force, ω = stream power, ω_U_ = unit stream power, ω_eff_ = effective stream power, Re = Reynolds number, τ_cr_ = critical shear stress. The complete dataset is presented in table S19.

### Quantification of different erosion processes

**Figure 3:**
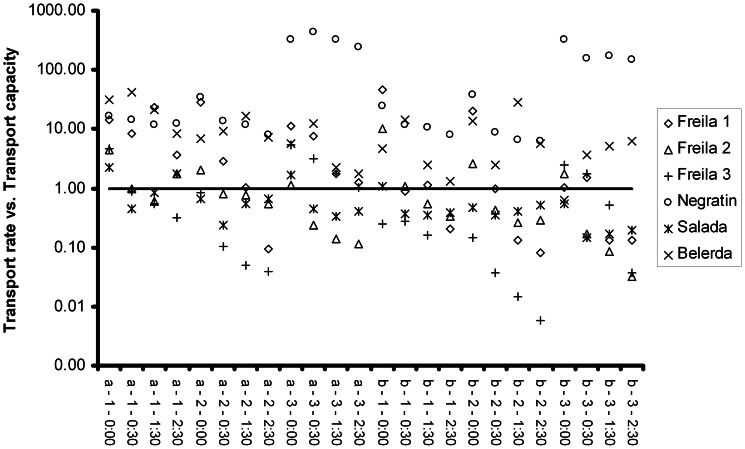
Transport rate vs. Transport capacity for each sample. The different experiments are represented by different symbols. On the x-axis, the following parameters are presented: run a or run b – measuring point 1–3 – sampling time at measuring point. The horizontal line marks the 1∶1-relation between transport rate and transport capacity.

Figure 3 shows the relationships between the measured transport rates and the predicted transport capacities. From 144 samples, in 82 cases the transport rate exceeds the capacity, corresponding to approximately 57% of all cases. Tables S20 and S21 present the differences between transport rates and transport capacities (S20) and the percentage of transport rate exceeding the capacity (S21) and hence the percentage of processes which are not controlled by the influence of shear stress. The percentage of material which is transported by processes independent of shear stress is on average 41.5% (see Table 5). Remarkably, the distribution is uneven, i.e. in the three Freila-experiments, the mean is 24.3% while in Negratin, Salada and Belerda, the average value is as high as 58.7% (see Table 5). The second group shows clearly higher sediment concentrations, meaning that the processes independent of shear stress provide higher sediment concentrations than the shear stress-based processes. This indicates that the influence of hydraulic parameters is higher for low sediment concentrations, or, in other words that high sediment concentrations are not caused by hydraulic parameters.

**Table 5:** Percentage of exceedance: Share of transport rate exceeding transport capacity.

Experiment	Value
Freila1	41.4%
Freila 2	16.0%
Freila 3	15.7%
Negratin	94.0%
Salada	6.0%
Belerda	76.1%
Average Freila 1–3	24.3%
Average Negratin, Salada, Belerda	58.7%
Average of all experiments	41.5%

In the first six rows, the exceedance percentage of each experiment is presented, in the next two rows the average percentage of the Freila experiments and of the Negratin-, Salada- and Belerda experiment and in the last row the average of all experiments. The complete dataset is presented in table S20.

## Discussion

A comparison with results of other research groups shows that the measured values are in a realistic range. Ghebreiyessus [3] measured shear stress values up to 40 Pa and in the experiments of Nearing et al. [4], Reynolds numbers of up to 100000 and unit stream power values of up to 10 m s^−1^ were reached. Giménez & Govers [5] found unit stream power values of up to 0.4 m s^−1^ and unit length shear force values of up to 6 N m^−1^. In a study of Zhang et al. [9], shear stress values of up to 30 Pa and unit stream power values of up to 0.5 m s^−1^ were reported. Govers [13] measured shear stress values of up to 100 Pa and effective stream power values of up to 10000 W m^−1^. While the measurements presented here are in the same order of magnitude compared to the previously published research, there are no clear linear correlations between hydraulic parameters and erosion parameters in the results of the field experiments. Therefore, these outcomes indicate that linear models may generally not be sufficient in order to describe the complex processes in natural rills.

Four possible improvements may help to improve this important concept which has been studied already for over thirty years, (1) including a clear description of the employed parameters, (2) including the turbulence, (3) considering the impact of processes that do not depend on the shear stress and likewise (4) consider the high spatial and temporal variability observed in natural rills. These potential improvements will be discussed in more detail below.

For instance, the flow shear stress, a hydraulic parameter, and the critical shear stress, a soil parameter (similar to soil strength), must be differentiated. In particular, the flow shear stress must exceed the critical shear stress for erosion to occur. A number of hydraulic parameters, such as the flow velocity or the fluid density, water depth or width and roughness are used for the computation of the flow shear stress. The actual version of the shear stress equation calculates the average shear stress by depth averaging of momentum equation for steady uniform flow per area and time. Some factors used in shear stress calculation have been developed from empirical studies [15]–[26]. In most cases, the theoretical basis of the equations is however not clear. The formula applied by Chisci et al. [27] is derived from Landau and Lifchitz [57]. Other versions of the Landau-Lifchitz equation can be found in the literature [2]–[5].

The critical shear stress is the force needed to detach a soil particle. So it corresponds to a soil parameter and therefore, input for calculations should also depend on soil characteristics. However, this is the case for the WEPP model [34] only, where the critical shear stress is calculated using soil parameters such as texture, organic matter content and dry bulk density. In other cases, both hydraulic and soil parameters are used [34]. The discrepancies in the methods of computation of the shear stress may be due to the conditions under which the equations are deduced, as these equations are based on empirical observations. The empirical nature of the development of the different expressions is clearly highlighted in previous work [30]–[32]. That means the equations are not deduced from physical laws but from empirical studies.

In many studies [12], [35], [37]–[41], neither critical shear stress nor shear stress are used for the calculation of the transport capacity at all. In other studies shear stress is used to calculate transport capacity and detachment capacity [36] or transport rate [42] and critical shear stress to calculate the detachment capacity [36]. In both cases it is clear that shear stress and critical shear stress operate against each other, the important parameter is the difference between these two variables.

A summary of these equations can be found in Reid and Dunne [58], on the EPA-homepage [59] and in Hessel and Jetten [60].

The second reason for the low R^2^- values in the correlations between hydraulic parameter and soil detachment can be the lack of turbulence parameters in the equations.

In the study of Knapen et al. [14] the Reynolds number shows very different correlations to the detachment rate, and this holds as well for the results of this study. The reason could be that the turbulence, described by the Reynolds number, does not directly operate on substrate, it influences the acting shear stress, that means the calculated shear stress is much lower than the operating shear stress, a relation which has been confirmed in several studies: Nearing et al. [61] found that turbulence can increase the active shear stress by a factor of several thousands. They measured flow shear stresses ranging from 0.5 to 2 Pa, and tensile strengths ranging from 1 to 2 kPa. Despite the fact that the tensile strengths are 1000 times larger than the flow shear stresses, the authors also measured detachment rates in the order of 300 g m^−2^ s^−1^. Such large detachment rates were attributed to turbulent burst events. Another study about the influence of turbulence on detachment rates was published by Nearing & Parker [62]. They showed that under turbulent flow conditions the same shear stress value caused a clearly higher detachment rate. In their flume experiments the difference between detachment rate caused by turbulent and laminar flow increased with increasing shear stress value, i.e., if given hydraulic conditions lead to a high shear stress value, the influence of turbulence on soil erosion is higher than in low shear stress value ranges.

The shear stress equation, as well as the equations describing other hydraulic parameters, assumes that drag forces are dominant for controlling erosion. But rill erosion is the result of the combination of different processes including headcut erosion, sidewall sloughing, tunnelling, micro-piping, slaking piping and sapping [14], [45], [63]–[67]. This is the third possible improvement for the problems of the model equations. The percentage of headcutting in the different studies ranges between “four times higher than the contribution of bed scours” [67] to “60% of total rill erosion” [68]. Stefanovic and Bryan [69] showed that concentrated flow causes sediment production primarily from knickpoints, chutes, meanders and bank failure. Govers [45] distinguished between hydraulic erosion, mass wasting processes on rill sidewalls, gullying and piping. Hydraulic rill erosion mostly occurred during three extreme runoff events. Mass wasting processes caused 37% of total erosion in rills. Gullying, the retreat erosion at knickpoints and headcuts caused about 12% of rill erosion rates. In the experiments presented here, the main mechanisms causing rill erosion were mass wasting and gullying processes, hence the correlations between hydraulic parameters and detachment rate are generally low. However, the hydraulic rill erosion only occurs in extreme runoff events, in most cases, the runoff values are too low to cause hydraulic rill erosion. The percentage of material which is transported independent of shear stress is very high on the water front samples. Here the transport of loose material is probably more important than in the other samples meaning that this process is mainly independent of shear stress. In these cases of transport rate vs. transport capacity <1 the independence of shear stress cannot be excluded, in the other cases the processes controlled by shear stress can occur. Thus, it can be deduced that, in the case of T_R_>T_C_, not only shear stress controlled processes provide the material; at least the difference between T_R_ and T_C_ is caused by processes independent of shear stress.

The experiments presented here show that the correlation between hydraulic parameters and detachment rate does neither change from one experiment to another, nor from one run to another, but from one measuring point and run to another. Thus, sediment producing processes have a high spatial and temporal variability. This is the fourth possible improvement for models. It is very difficult to propose a single factor that always describes the soil detachment satisfactory. The high variability of erosion processes, even under controlled experimental conditions, has been highlighted in different studies. Measured variability shows a wide range between 3.4% and 173.2% [70]–[75]. This is partially the result of non-homogeneous parameters concerning soil characteristics and rainfall. On experimental plots, infiltration rates and soil aggregate stability can be highly variable [76] and rainfall also shows a high spatial and temporal variability [77]. Therefore, the input parameters to the different measurements reflected in the mentioned studies were not really comparable. Nevertheless, the results also make clear that modelling soil erosion has to include uncertainty in model input, as well as in the data used for model calibration and validation.

In field experiments, the spatial and temporal variability of soil conditions cannot be avoided, and is, furthermore, part of the investigations. Thus, additional input parameters as rainfall or flow should be maintained constant in the experiments to generate reproducible data. The high variability in soil erosion processes cannot be represented by a single factor like shear stress.

The results show that there is not a simple linear correlation between a certain hydraulic parameter and soil detachment rate. Depending on model purpose and scale, the factors can be used to predict the magnitude of rill detachment but they are not applicable for the simulation of rill erosion with high-resolution spatial and temporal change in processes.

A newer approach is the use of probability density functions to predict soil detachment [78], [79]. Sidorchuk gives two sources of stochasticity in erosion modelling: (1) the necessity of spatial and temporal averaging when determining deterministic equations, which describe concentrated flow erosion and (2) the fact that the main erosion factors, if these can be determined anyway, can only be measured with limited accuracy. This is not the first attempt to model erosion by relating the probability of soil detachment with the excess of erosion driving forces over soil erosion resistance forces, other articles using a stochastic approach to describe soil erosion were published by Nearing [80], Wilson [81] and Sidorchuk [82]–[87]. Notably, one of the earliest articles about stochastic in erosion processes has been published by Einstein [88]. These stochastic models reduce the number of empirical components. Applying these models to the experiments presented here is beyond the scope of the current study.

## Conclusions

The results show that a linear correlation between hydraulic parameter and soil detachment is not sufficient to describe processes in natural rills. The reason for this behaviour is the combination of various processes that can cause different amounts of soil erosion. The shear stress, for instance, only describes one process, while the results clearly show that there is not one fixed parameter that always predicts soil detachment best. Applicability of one certain hydraulic parameter to predict the sediment concentration changes at a certain point in time within a few minutes, because the temporal and spatial distribution of the different erosion processes is highly randomly determined. Therefore, it might be more useful to formulate results in probabilistic terms, an approach which has already been implemented by previous researchers, but is beyond the current work.

## Supporting Information

Table S1 **Freila 1 erosion data.**
(DOC)

Table S2 **Freila 1 runoff data.**
(DOC)

Table S3 **Freila 1 hydraulic data.**
(DOC)

Table S4 **Freila 2 erosion data.**
(DOC)

Table S5 **Freila 2 runoff data.**
(DOC)

Table S6 **Freila 2 hydraulic data.**
(DOC)

Table S7 **Freila 3 erosion data.**
(DOC)

Table S8 **Freila 3 runoff data.**
(DOC)

Table S9 **Freila 3 hydraulic data.**
(DOC)

Table S10 **Negratin erosion data.**
(DOC)

Table S11 **Negratin runoff data.**
(DOC)

Table S12 **Negratin hydraulic data.**
(DOC)

Table S13 **Salada erosion data.**
(DOC)

Table S14 **Salada runoff data.**
(DOC)

Table S15 **Salada hydraulic data.**
(DOC)

Table S16 **Belerda erosion data.**
(DOC)

Table S17 **Belerda runoff data.**
(DOC)

Table S18 **Belerda hydraulic data.**
(DOC)

Table S19 **R^2^ - values between the detachment rate and different hydraulic parameters.**
(DOC)

Table S20 **Comparison of the transport rate with the transport capacity: Transport rate T_R_ [kg s^−1^] - Transport capacity T_C_ [kg s^−1^].**
(DOC)

Table S21 **Comparison of the transport rate with the transport capacity: Percentage of T_R_ exceeding T_C_.**
(DOC)
