# Supplementary material for: Applicability of Different Hydraulic Parameters to Describe Soil Detachment in Eroding Rills
Source: PLoS One. 2013 May 24;8(5):e64861. doi: 10.1371/journal.pone.0064861 (PMC3663750; doi:10.1371/journal.pone.0064861)
Supplement: Table S1 — Freila 1 erosion data. (DOC) [file pone.0064861.s001.doc]

Table S1 Freila 1 erosion data

| Run - MP - flow length [m]- sampling time [min:sec] | Sediment Concentration [g L^-1^] | Detachment rate [kg s^-1^ m^-2^] | Transport rate [kg s^-1^] | Sample density [g cm^-3^] | Slope [°] | Transport capacity [kg s^-1^] |
| --- | --- | --- | --- | --- | --- | --- |
| a-1-3.4-0:00 | 10.6 | 0.0156 | 0.017750523 | 1.01 | 2.4 | 0.00125 |
| a-1-3.4-0:30 | 2.1 | 0.0092 | 0.010500304 | 1.00 | 2.4 | 0.00128 |
| a-1-3.4-1:30 | 3.3 | 0.0257 | 0.029404312 | 1.00 | 2.4 | 0.00129 |
| a-1-3.4-2:30 | 0.4 | 0.0040 | 0.004617795 | 1.00 | 2.4 | 0.00126 |
| a-2-8.6-0:00 | 14.9 | 0.0008 | 0.000726251 | 1.01 | 7.4 | 0.00003 |
| a-2-8.6-0:30 | 2.4 | 0.0004 | 0.000914653 | 1.00 | 7.4 | 0.00032 |
| a-2-8.6-1:30 | 1.4 | 0.0005 | 0.001633948 | 1.00 | 7.4 | 0.00156 |
| a-2-8.6-2:30 | 0.6 | 0.0010 | 0.007309575 | 1.00 | 7.4 | 0.07782 |
| a-3-13.1-0:00 | 26.6 | 0.0407 | 0.240254717 | 1.02 | 6 | 0.02132 |
| a-3-13.1-0:30 | 8.0 | 0.0079 | 0.041958790 | 1.01 | 6 | 0.00555 |
| a-3-13.1-1:30 | 3.1 | 0.0049 | 0.028349123 | 1.00 | 6 | 0.01600 |
| a-3-13.1-2:30 | 2.5 | 0.0042 | 0.024596630 | 1.00 | 6 | 0.01957 |
| b-1-3.4-0:00 | 35.1 | 0.0524 | 0.060019839 | 1.02 | 2.4 | 0.00132 |
| b-1-3.4-0:30 | 0.5 | 0.0010 | 0.001109306 | 1.00 | 2.4 | 0.00126 |
| b-1-3.4-1:30 | 0.6 | 0.0012 | 0.001397038 | 1.00 | 2.4 | 0.00126 |
| b-1-3.4-2:30 | 0.1 | 0.0002 | 0.000302770 | 1.00 | 2.4 | 0.00144 |
| b-2-8.6-0:00 | 4.8 | 0.0001 | 0.000126626 | 1.00 | 7.4 | 0.00001 |
| b-2-8.6-0:30 | 0.6 | 0.0001 | 0.000260038 | 1.00 | 7.4 | 0.00027 |
| b-2-8.6-1:30 | 0.7 | 0.0013 | 0.010225583 | 1.00 | 7.4 | 0.07834 |
| b-2-8.6-2:30 | 0.1 | 0.0000 | 0.000132974 | 1.00 | 7.4 | 0.00161 |
| b-3-13.1-0:00 | 9.9 | 0.0336 | 0.247153306 | 1.01 | 6 | 0.24591 |
| b-3-13.1-0:30 | 2.6 | 0.0021 | 0.012025713 | 1.00 | 6 | 0.00812 |
| b-3-13.1-1:30 | 1.0 | 0.0042 | 0.030456868 | 1.00 | 6 | 0.22937 |
| b-3-13.1-2:30 | 0.8 | 0.0047 | 0.035191499 | 1.00 | 6 | 0.26525 |
