# Supplementary material for: Applicability of Different Hydraulic Parameters to Describe Soil Detachment in Eroding Rills
Source: PLoS One. 2013 May 24;8(5):e64861. doi: 10.1371/journal.pone.0064861 (PMC3663750; doi:10.1371/journal.pone.0064861)
Supplement: Table S2 — Freila 1 runoff data. (DOC) [file pone.0064861.s002.doc]

Table S2 Freila 1 runoff data

| Run - MP - flow length [m]- sampling time [min:sec] | Flow velocity [m s^-1^] | Dynamic viscosity [kg s^-1^ m^-1^] | Water depth [cm] | Flow cross section [cm²] | Wetted Perimeter [cm] | Hydraulic radius [cm] |
| --- | --- | --- | --- | --- | --- | --- |
| a-1-3.4-0:00 | 0.41 | 0.001053 | 0.20 | 40.9 | 33.5 | 1.2 |
| a-1-3.4-0:30 | 1.20 | 0.001011 | 0.30 | 41.7 | 33.7 | 1.2 |
| a-1-3.4-1:30 | 2.12 | 0.001017 | 0.30 | 41.7 | 33.7 | 1.2 |
| a-1-3.4-2:30 | 2.61 | 0.001002 | 0.40 | 42.0 | 34.2 | 1.2 |
| a-2-8.6-0:00 | 0.34 | 0.001075 | 0.30 | 1.4 | 10.9 | 0.1 |
| a-2-8.6-0:30 | 0.39 | 0.001012 | 1.30 | 10.0 | 27.6 | 0.4 |
| a-2-8.6-1:30 | 0.41 | 0.001007 | 2.50 | 28.0 | 41.1 | 0.7 |
| a-2-8.6-2:30 | 0.41 | 0.001003 | 10.30 | 289.7 | 88.9 | 3.3 |
| a-3-13.1-0:00 | 0.92 | 0.001133 | 3.60 | 98.3 | 45.1 | 2.2 |
| a-3-13.1-0:30 | 1.01 | 0.001040 | 2.40 | 51.8 | 40.5 | 1.3 |
| a-3-13.1-1:30 | 1.05 | 0.001016 | 3.10 | 86.9 | 44.3 | 2.0 |
| a-3-13.1-2:30 | 1.06 | 0.001012 | 3.30 | 94.9 | 44.6 | 2.1 |
| b-1-3.4-0:00 | 0.41 | 0.001176 | 0.30 | 41.7 | 33.7 | 1.2 |
| b-1-3.4-0:30 | 0.57 | 0.001002 | 0.40 | 42.0 | 34.2 | 1.2 |
| b-1-3.4-1:30 | 0.59 | 0.001003 | 0.40 | 42.0 | 34.2 | 1.2 |
| b-1-3.4-2:30 | 0.91 | 0.001000 | 0.50 | 47.2 | 36.3 | 1.3 |
| b-2-8.6-0:00 | 0.32 | 0.001024 | 0.50 | 0.8 | 10.9 | 0.1 |
| b-2-8.6-0:30 | 0.49 | 0.001003 | 1.30 | 9.4 | 28.0 | 0.3 |
| b-2-8.6-1:30 | 0.53 | 0.001003 | 10.40 | 290.7 | 89.0 | 3.3 |
| b-2-8.6-2:30 | 0.46 | 0.001001 | 2.40 | 26.1 | 37.7 | 0.7 |
| b-3-13.1-0:00 | 0.76 | 0.001050 | 9.20 | 327.5 | 56.2 | 5.8 |
| b-3-13.1-0:30 | 0.72 | 0.001013 | 2.80 | 64.0 | 42.8 | 1.5 |
| b-3-13.1-1:30 | 0.98 | 0.001005 | 9.20 | 318.0 | 55.9 | 5.7 |
| b-3-13.1-2:30 | 1.32 | 0.001004 | 9.30 | 346.0 | 57.4 | 6.0 |
