# Supplementary material for: Applicability of Different Hydraulic Parameters to Describe Soil Detachment in Eroding Rills
Source: PLoS One. 2013 May 24;8(5):e64861. doi: 10.1371/journal.pone.0064861 (PMC3663750; doi:10.1371/journal.pone.0064861)
Supplement: Table S3 — Freila 1 hydraulic data. (DOC) [file pone.0064861.s003.doc]

Table S3 Freila 1 hydraulic data

| Run - MP - flow length [m]- sampling time [min:sec] | τ [Pa] | Г [N m^-1^] | ω [W m^-2^] | ω_U_ [m s^-1^] | ω_eff_ [W m^-1^] | Re [ ] | τ - τ_cr_ [Pa] |
| --- | --- | --- | --- | --- | --- | --- | --- |
| a-1-3.4-0:00 | 5.05 | 1.69 | 2.07 | 0.02 | 187.91 | 4791 | 3.08 |
| a-1-3.4-0:30 | 5.09 | 1.72 | 6.10 | 0.05 | 725.17 | 14707 | 3.12 |
| a-1-3.4-1:30 | 5.09 | 1.72 | 10.79 | 0.09 | 1703.78 | 25834 | 3.12 |
| a-1-3.4-2:30 | 5.05 | 1.73 | 13.18 | 0.11 | 1898.77 | 32015 | 3.08 |
| a-2-8.6-0:00 | 1.68 | 0.18 | 0.57 | 0.04 | 20.68 | 420 | -0.30 |
| a-2-8.6-0:30 | 4.58 | 1.26 | 1.77 | 0.05 | 42.55 | 1384 | 2.61 |
| a-2-8.6-1:30 | 8.62 | 3.54 | 3.53 | 0.05 | 77.65 | 2776 | 6.64 |
| a-2-8.6-2:30 | 41.19 | 36.61 | 16.89 | 0.05 | 315.87 | 13326 | 39.22 |
| a-3-13.1-0:00 | 22.72 | 10.25 | 20.90 | 0.10 | 876.60 | 17996 | 20.75 |
| a-3-13.1-0:30 | 13.20 | 5.34 | 13.31 | 0.11 | 583.29 | 12474 | 11.23 |
| a-3-13.1-1:30 | 20.14 | 8.93 | 21.11 | 0.11 | 982.55 | 20267 | 18.17 |
| a-3-13.1-2:30 | 21.83 | 9.74 | 23.08 | 0.11 | 1077.88 | 22237 | 19.86 |
| b-1-3.4-0:00 | 5.20 | 1.75 | 2.13 | 0.02 | 149.50 | 4412 | 3.23 |
| b-1-3.4-0:30 | 5.05 | 1.73 | 2.90 | 0.02 | 196.40 | 7053 | 3.08 |
| b-1-3.4-1:30 | 5.05 | 1.73 | 2.98 | 0.02 | 204.64 | 7245 | 3.08 |
| b-1-3.4-2:30 | 5.33 | 1.94 | 4.84 | 0.04 | 363.78 | 11770 | 3.36 |
| b-2-8.6-0:00 | 0.96 | 0.10 | 0.31 | 0.04 | 5.81 | 237 | -1.01 |
| b-2-8.6-0:30 | 4.25 | 1.19 | 2.08 | 0.06 | 54.38 | 1644 | 2.28 |
| b-2-8.6-1:30 | 41.30 | 36.75 | 21.83 | 0.07 | 461.19 | 17220 | 39.33 |
| b-2-8.6-2:30 | 8.73 | 3.29 | 4.00 | 0.06 | 96.25 | 3166 | 6.76 |
| b-3-13.1-0:00 | 60.18 | 33.79 | 45.74 | 0.08 | 1517.71 | 42492 | 58.21 |
| b-3-13.1-0:30 | 15.35 | 6.58 | 11.01 | 0.08 | 396.41 | 10602 | 13.38 |
| b-3-13.1-1:30 | 58.39 | 32.63 | 56.94 | 0.10 | 2108.18 | 55255 | 56.42 |
| b-3-13.1-2:30 | 61.89 | 35.49 | 81.83 | 0.14 | 3606.26 | 79497 | 59.92 |
