# Supplementary material for: Applicability of Different Hydraulic Parameters to Describe Soil Detachment in Eroding Rills
Source: PLoS One. 2013 May 24;8(5):e64861. doi: 10.1371/journal.pone.0064861 (PMC3663750; doi:10.1371/journal.pone.0064861)
Supplement: Table S4 — Freila 2 erosion data. (DOC) [file pone.0064861.s004.doc]

Table S4 Freila 2 erosion data

| Run - MP - flow length [m]- sampling time [min:sec] | Sediment Concentration [g L^-1^] | Detachment rate [kg s^-1^ m^-2^] | Transport rate [kg s^-1^] | Sample density [g cm^-3^] | Slope [°] | Transport capacity [kg s^-1^] |
| --- | --- | --- | --- | --- | --- | --- |
| a-1-4-0:00 | 16.8 | 0.0668 | 0.267274644 | 1.01 | 5.7 | 0.05891 |
| a-1-4-0:30 | 2.4 | 0.0078 | 0.023207900 | 1.00 | 5.7 | 0.02325 |
| a-1-4-1:30 | 1.4 | 0.0058 | 0.014046683 | 1.00 | 5.7 | 0.02362 |
| a-1-4-2:30 | 2.3 | 0.0086 | 0.019756646 | 1.00 | 5.7 | 0.01134 |
| a-2-8.5-0:00 | 11.3 | 0.0079 | 0.033779926 | 1.01 | 3.4 | 0.01692 |
| a-2-8.5-0:30 | 3.9 | 0.0033 | 0.014486969 | 1.00 | 3.4 | 0.01837 |
| a-2-8.5-1:30 | 2.8 | 0.0028 | 0.011787666 | 1.00 | 3.4 | 0.01570 |
| a-2-8.5-2:30 | 1.8 | 0.0023 | 0.010364921 | 1.00 | 3.4 | 0.01946 |
| a-3-13.3-0:00 | 19.2 | 0.0239 | 0.287705065 | 1.01 | 7.4 | 0.25306 |
| a-3-13.3-0:30 | 6.1 | 0.0127 | 0.181365395 | 1.00 | 7.4 | 0.77566 |
| a-3-13.3-1:30 | 3.1 | 0.0070 | 0.098724460 | 1.00 | 7.4 | 0.71743 |
| a-3-13.3-2:30 | 2.3 | 0.0057 | 0.079795433 | 1.00 | 7.4 | 0.71687 |
| b-1-4-0:00 | 12.4 | 0.0193 | 0.037281867 | 1.01 | 5.7 | 0.00362 |
| b-1-4-0:30 | 2.0 | 0.0062 | 0.014347225 | 1.00 | 5.7 | 0.01351 |
| b-1-4-1:30 | 0.8 | 0.0027 | 0.006088968 | 1.00 | 5.7 | 0.01132 |
| b-1-4-2:30 | 0.4 | 0.0017 | 0.003861665 | 1.00 | 5.7 | 0.01132 |
| b-2-8.5-0:00 | 12.0 | 0.0103 | 0.044903275 | 1.01 | 3.4 | 0.01704 |
| b-2-8.5-0:30 | 1.6 | 0.0018 | 0.008409765 | 1.00 | 3.4 | 0.01955 |
| b-2-8.5-1:30 | 0.8 | 0.0010 | 0.005012019 | 1.00 | 3.4 | 0.01954 |
| b-2-8.5-2:30 | 0.7 | 0.0012 | 0.005702665 | 1.00 | 3.4 | 0.01954 |
| b-3-13.3-0:00 | 38.0 | 0.0371 | 0.445794531 | 1.02 | 7.4 | 0.25751 |
| b-3-13.3-0:30 | 2.9 | 0.0092 | 0.132228769 | 1.00 | 7.4 | 0.77333 |
| b-3-13.3-1:30 | 1.6 | 0.0042 | 0.059490319 | 1.00 | 7.4 | 0.71636 |
| b-3-13.3-2:30 | 1.4 | 0.0016 | 0.022511209 | 1.00 | 7.4 | 0.71624 |
