# Supplementary material for: Applicability of Different Hydraulic Parameters to Describe Soil Detachment in Eroding Rills
Source: PLoS One. 2013 May 24;8(5):e64861. doi: 10.1371/journal.pone.0064861 (PMC3663750; doi:10.1371/journal.pone.0064861)
Supplement: Table S5 — Freila 2 runoff data. (DOC) [file pone.0064861.s005.doc]

Table S5 Freila 2 runoff data

| Run - MP - flow length [m]- sampling time [min:sec] | Flow velocity [m s^-1^] | Dynamic viscosity [kg s^-1^ m^-1^] | Water depth [cm] | Flow cross section [cm²] | Wetted Perimeter [cm] | Hydraulic radius [cm] |
| --- | --- | --- | --- | --- | --- | --- |
| a-1-4-0:00 | 0.47 | 0.001084 | 4.7 | 338.62 | 99.98 | 3.39 |
| a-1-4-0:30 | 0.54 | 0.001012 | 2.8 | 174.04 | 74.13 | 2.35 |
| a-1-4-1:30 | 0.69 | 0.001007 | 2.4 | 142.28 | 60.20 | 2.36 |
| a-1-4-2:30 | 0.84 | 0.001012 | 1.6 | 100.62 | 57.12 | 1.76 |
| a-2-8.5-0:00 | 0.21 | 0.001057 | 0.3 | 142.06 | 50.60 | 2.81 |
| a-2-8.5-0:30 | 0.24 | 0.001020 | 0.5 | 152.10 | 52.27 | 2.91 |
| a-2-8.5-1:30 | 0.31 | 0.001014 | 0.2 | 134.99 | 49.38 | 2.73 |
| a-2-8.5-2:30 | 0.38 | 0.001009 | 0.5 | 152.10 | 52.27 | 2.98 |
| a-3-13.3-0:00 | 0.32 | 0.001096 | 4.2 | 469.04 | 90.43 | 5.19 |
| a-3-13.3-0:30 | 0.34 | 0.001031 | 8.2 | 877.69 | 107.58 | 8.16 |
| a-3-13.3-1:30 | 0.37 | 0.001016 | 8 | 838.70 | 105.94 | 7.92 |
| a-3-13.3-2:30 | 0.41 | 0.001012 | 8 | 838.70 | 105.94 | 7.92 |
| b-1-4-0:00 | 0.56 | 0.001062 | 0.8 | 53.72 | 48.34 | 1.11 |
| b-1-4-0:30 | 0.67 | 0.001010 | 1.8 | 109.04 | 57.70 | 1.89 |
| b-1-4-1:30 | 0.80 | 0.001004 | 1.6 | 100.62 | 57.12 | 1.76 |
| b-1-4-2:30 | 0.87 | 0.001002 | 1.6 | 100.62 | 57.12 | 1.76 |
| b-2-8.5-0:00 | 0.26 | 0.001060 | 0.4 | 143.66 | 51.04 | 2.81 |
| b-2-8.5-0:30 | 0.31 | 0.001008 | 0.8 | 168.31 | 56.37 | 2.99 |
| b-2-8.5-1:30 | 0.39 | 0.001004 | 0.8 | 168.31 | 56.37 | 2.99 |
| b-2-8.5-2:30 | 0.48 | 0.001004 | 0.8 | 168.31 | 56.37 | 2.99 |
| b-3-13.3-0:00 | 0.25 | 0.001190 | 4.2 | 469.04 | 90.43 | 5.19 |
| b-3-13.3-0:30 | 0.52 | 0.001015 | 8.2 | 877.69 | 107.58 | 8.16 |
| b-3-13.3-1:30 | 0.45 | 0.001008 | 8 | 838.70 | 105.94 | 7.92 |
| b-3-13.3-2:30 | 0.19 | 0.001007 | 8 | 838.70 | 105.94 | 7.92 |
