# Supplementary material for: Applicability of Different Hydraulic Parameters to Describe Soil Detachment in Eroding Rills
Source: PLoS One. 2013 May 24;8(5):e64861. doi: 10.1371/journal.pone.0064861 (PMC3663750; doi:10.1371/journal.pone.0064861)
Supplement: Table S6 — Freila 2 hydraulic data. (DOC) [file pone.0064861.s006.doc]

Table S6 Freila 2 hydraulic data

| Run - MP - flow length [m]- sampling time [min:sec] | τ [Pa] | Г [N m^-1^] | ω [W m^-2^] | ω_U_ [m s^-1^] | ω_eff_ [W m^-1^] | Re [ ] | τ - τ_cr_ [Pa] |
| --- | --- | --- | --- | --- | --- | --- | --- |
| a-1-4-0:00 | 33.35 | 33.34 | 15.67 | 0.05 | 476.44 | 14840.13 | 31.28 |
| a-1-4-0:30 | 22.91 | 16.98 | 12.47 | 0.05 | 477.57 | 12643.85 | 20.84 |
| a-1-4-1:30 | 23.05 | 13.87 | 15.97 | 0.07 | 767.17 | 16276.69 | 20.98 |
| a-1-4-2:30 | 17.19 | 9.82 | 14.47 | 0.08 | 866.60 | 14676.93 | 15.12 |
| a-2-8.5-0:00 | 16.45 | 8.32 | 3.45 | 0.01 | 308.68 | 5619.61 | 14.38 |
| a-2-8.5-0:30 | 16.97 | 8.87 | 4.14 | 0.01 | 287.89 | 6976.46 | 14.90 |
| a-2-8.5-1:30 | 15.93 | 7.87 | 4.96 | 0.02 | 696.51 | 8412.52 | 13.86 |
| a-2-8.5-2:30 | 17.36 | 9.07 | 6.58 | 0.02 | 577.49 | 11212.34 | 15.29 |
| a-3-13.3-0:00 | 66.33 | 59.98 | 21.22 | 0.04 | 809.25 | 15329.03 | 64.26 |
| a-3-13.3-0:30 | 103.48 | 111.32 | 34.97 | 0.04 | 1095.81 | 26859.14 | 101.41 |
| a-3-13.3-1:30 | 100.22 | 106.18 | 37.48 | 0.05 | 1235.77 | 29202.70 | 98.15 |
| a-3-13.3-2:30 | 100.17 | 106.12 | 41.06 | 0.05 | 1417.16 | 32125.42 | 98.10 |
| b-1-4-0:00 | 10.91 | 5.27 | 6.11 | 0.06 | 377.59 | 5905.24 | 8.84 |
| b-1-4-0:30 | 18.44 | 10.64 | 12.30 | 0.07 | 628.24 | 12503.04 | 16.37 |
| b-1-4-1:30 | 17.17 | 9.81 | 13.73 | 0.08 | 801.27 | 14039.07 | 15.10 |
| b-1-4-2:30 | 17.17 | 9.81 | 14.92 | 0.09 | 908.07 | 15284.39 | 15.10 |
| b-2-8.5-0:00 | 16.50 | 8.42 | 4.29 | 0.02 | 352.57 | 6954.96 | 14.43 |
| b-2-8.5-0:30 | 17.39 | 9.80 | 5.34 | 0.02 | 308.90 | 9112.39 | 15.32 |
| b-2-8.5-1:30 | 17.38 | 9.80 | 6.84 | 0.02 | 447.03 | 11708.97 | 15.31 |
| b-2-8.5-2:30 | 17.38 | 9.80 | 8.25 | 0.03 | 592.94 | 14138.38 | 15.31 |
| b-3-13.3-0:00 | 67.10 | 60.68 | 16.78 | 0.03 | 568.65 | 11156.44 | 65.03 |
| b-3-13.3-0:30 | 103.27 | 111.10 | 53.49 | 0.07 | 2072.79 | 41729.93 | 101.20 |
| b-3-13.3-1:30 | 100.12 | 106.07 | 45.47 | 0.06 | 1651.29 | 35706.89 | 98.05 |
| b-3-13.3-2:30 | 100.11 | 106.06 | 19.44 | 0.03 | 461.72 | 15281.78 | 98.04 |
