# Supplementary material for: Applicability of Different Hydraulic Parameters to Describe Soil Detachment in Eroding Rills
Source: PLoS One. 2013 May 24;8(5):e64861. doi: 10.1371/journal.pone.0064861 (PMC3663750; doi:10.1371/journal.pone.0064861)
Supplement: Table S7 — Freila 3 erosion data. (DOC) [file pone.0064861.s007.doc]

Table S7 Freila 3 erosion data

| Run - MP - flow length [m]- sampling time [min:sec] | Sediment Concentration [g L^-1^] | Detachment rate  [kg s^-1^ m^-2^] | Transport rate [kg s^-1^] | Sample density  [g cm^-3^] | Slope [°] | Transport capacity [kg s^-1^] |
| --- | --- | --- | --- | --- | --- | --- |
| a-1-2.9-0:00 | 45.0 | 0.2958 | 0.379845863 | 1.03 | 4.7 | 0.08011 |
| a-1-2.9-0:30 | 3.0 | 0.0174 | 0.017769517 | 1.00 | 4.7 | 0.02040 |
| a-1-2.9-1:30 | 1.6 | 0.0135 | 0.014059196 | 1.00 | 4.7 | 0.02558 |
| a-1-2.9-2:30 | 0.8 | 0.0081 | 0.008537001 | 1.00 | 4.7 | 0.02670 |
| a-2-11-0:00 | 43.2 | 0.1264 | 0.653849085 | 1.03 | 15.1 | 0.77604 |
| a-2-11-0:30 | 7.8 | 0.0298 | 0.189057846 | 1.00 | 15.1 | 1.85097 |
| a-2-11-1:30 | 4.3 | 0.0173 | 0.117480752 | 1.00 | 15.1 | 2.37381 |
| a-2-11-2:30 | 3.7 | 0.0161 | 0.120276643 | 1.00 | 15.1 | 3.15647 |
| a-3-13.8-0:00 | 56.3 | 0.0619 | 0.414999121 | 1.04 | 4.4 | 0.07706 |
| a-3-13.8-0:30 | 11.1 | 0.0144 | 0.087141739 | 1.01 | 4.4 | 0.02722 |
| a-3-13.8-1:30 | 5.5 | 0.0080 | 0.048446063 | 1.00 | 4.4 | 0.02707 |
| a-3-13.8-2:30 | 3.9 | 0.0068 | 0.043053290 | 1.00 | 4.4 | 0.04278 |
| b-1-2.9-0:00 | 3.5 | 0.0257 | 0.036744778 | 1.00 | 4.7 | 0.14627 |
| b-1-2.9-0:30 | 1.4 | 0.0178 | 0.023345189 | 1.00 | 4.7 | 0.08610 |
| b-1-2.9-1:30 | 0.4 | 0.0054 | 0.006318732 | 1.00 | 4.7 | 0.03960 |
| b-1-2.9-2:30 | 0.0 | 0.0000 | 0.000000000 | 1.00 | 4.7 | 0.03846 |
| b-2-11-0:00 | 10.2 | 0.0422 | 0.267669423 | 1.01 | 15.1 | 1.85510 |
| b-2-11-0:30 | 3.3 | 0.0122 | 0.080111940 | 1.00 | 15.1 | 2.12315 |
| b-2-11-1:30 | 1.9 | 0.0055 | 0.038627457 | 1.00 | 15.1 | 2.57786 |
| b-2-11-2:30 | 1.2 | 0.0026 | 0.019920464 | 1.00 | 15.1 | 3.37596 |
| b-3-13.8-0:00 | 26.1 | 0.0421 | 0.302333459 | 1.02 | 4.4 | 0.12407 |
| b-3-13.8-0:30 | 6.1 | 0.0121 | 0.076629194 | 1.00 | 4.4 | 0.04286 |
| b-3-13.8-1:30 | 2.5 | 0.0034 | 0.021755547 | 1.00 | 4.4 | 0.04272 |
| b-3-13.8-2:30 | 2.2 | 0.0002 | 0.001559311 | 1.00 | 4.4 | 0.04271 |
