# Supplementary material for: Applicability of Different Hydraulic Parameters to Describe Soil Detachment in Eroding Rills
Source: PLoS One. 2013 May 24;8(5):e64861. doi: 10.1371/journal.pone.0064861 (PMC3663750; doi:10.1371/journal.pone.0064861)
Supplement: Table S8 — Freila 3 runoff data. (DOC) [file pone.0064861.s008.doc]

Table S8 Freila 3 runoff data

| Run - MP - flow length [m]- sampling time [min:sec] | Flow velocity [m s^-1^] | Dynamic viscosity [kg s^-1^ m^-1^] | Water depth [cm] | Flow cross section [cm²] | Wetted Perimeter [cm] | Hydraulic radius [cm] |
| --- | --- | --- | --- | --- | --- | --- |
| a-1-2.9-0:00 | 0.44 | 0.001225 | 7.6 | 191.68 | 45.06 | 4.25 |
| a-1-2.9-0:30 | 0.65 | 0.001015 | 4.3 | 89.43 | 35.77 | 2.50 |
| a-1-2.9-1:30 | 0.90 | 0.001008 | 4.8 | 100.07 | 36.54 | 2.74 |
| a-1-2.9-2:30 | 1.00 | 0.001004 | 4.9 | 103.52 | 37.15 | 2.79 |
| a-2-11-0:00 | 0.61 | 0.001216 | 10.00 | 248.00 | 47.02 | 5.27 |
| a-2-11-0:30 | 0.55 | 0.001039 | 15.00 | 436.45 | 57.68 | 7.57 |
| a-2-11-1:30 | 0.52 | 0.001022 | 17.00 | 517.84 | 61.88 | 8.37 |
| a-2-11-2:30 | 0.51 | 0.001018 | 20.00 | 638.72 | 68.08 | 9.38 |
| a-3-13.8-0:00 | 0.35 | 0.001281 | 7.00 | 210.67 | 48.55 | 4.34 |
| a-3-13.8-0:30 | 0.62 | 0.001055 | 5.00 | 128.02 | 44.00 | 2.91 |
| a-3-13.8-1:30 | 0.69 | 0.001027 | 5.00 | 128.02 | 44.00 | 2.91 |
| a-3-13.8-2:30 | 0.68 | 0.001020 | 6.00 | 160.05 | 45.78 | 3.50 |
| b-1-2.9-0:00 | 0.38 | 0.001018 | 9.7 | 276.10 | 50.23 | 5.50 |
| b-1-2.9-0:30 | 0.82 | 0.001007 | 5.9 | 204.80 | 46.02 | 4.45 |
| b-1-2.9-1:30 | 1.33 | 0.001002 | 5.7 | 134.37 | 41.18 | 3.26 |
| b-1-2.9-2:30 | 1.55 | 0.001000 | 5.6 | 123.89 | 38.41 | 3.23 |
| b-2-11-0:00 | 0.6 | 0.001051 | 15.00 | 436.45 | 57.68 | 7.57 |
| b-2-11-0:30 | 0.51 | 0.001016 | 16.00 | 479.77 | 59.92 | 8.01 |
| b-2-11-1:30 | 0.38 | 0.001009 | 18.00 | 550.13 | 63.54 | 8.66 |
| b-2-11-2:30 | 0.25 | 0.001006 | 21.00 | 674.83 | 69.96 | 9.65 |
| b-3-13.8-0:00 | 0.42 | 0.001130 | 9.00 | 276.30 | 52.05 | 5.31 |
| b-3-13.8-0:30 | 0.78 | 0.001031 | 6.00 | 160.05 | 45.78 | 3.50 |
| b-3-13.8-1:30 | 0.54 | 0.001013 | 6.00 | 160.05 | 45.78 | 3.50 |
| b-3-13.8-2:30 | 0.04 | 0.001011 | 6.00 | 160.05 | 45.78 | 3.50 |
