# Supplementary material for: Applicability of Different Hydraulic Parameters to Describe Soil Detachment in Eroding Rills
Source: PLoS One. 2013 May 24;8(5):e64861. doi: 10.1371/journal.pone.0064861 (PMC3663750; doi:10.1371/journal.pone.0064861)
Supplement: Table S9 — Freila 3 hydraulic data. (DOC) [file pone.0064861.s009.doc]

Table S9 Freila 3 hydraulic data

| Run - MP - flow length [m]- sampling time [min:sec] | τ [Pa] | Г [N m^-1^] | ω [W m^-2^] | ω_U_ [m s^-1^] | ω_eff_ [W m^-1^] | Re [ ] | τ - τ_cr_ [Pa] |
| --- | --- | --- | --- | --- | --- | --- | --- |
| a-1-2.9-0:00 | 35.16 | 15.84 | 15.47 | 0.04 | 339.16 | 15709.50 | 33.19 |
| a-1-2.9-0:30 | 20.13 | 7.20 | 13.13 | 0.05 | 387.49 | 16085.57 | 18.16 |
| a-1-2.9-1:30 | 22.03 | 8.05 | 19.74 | 0.07 | 664.13 | 24368.99 | 20.06 |
| a-1-2.9-2:30 | 22.41 | 8.33 | 22.33 | 0.08 | 788.02 | 27665.64 | 20.44 |
| a-2-11-0:00 | 138.45 | 65.10 | 84.45 | 0.16 | 3602.41 | 27174.42 | 136.48 |
| a-2-11-0:30 | 194.31 | 112.09 | 107.33 | 0.14 | 3938.82 | 40414.91 | 192.34 |
| a-2-11-1:30 | 214.45 | 132.70 | 111.95 | 0.14 | 3859.87 | 42875.13 | 212.48 |
| a-2-11-2:30 | 240.30 | 163.61 | 123.07 | 0.13 | 3992.08 | 47287.94 | 238.33 |
| a-3-13.8-0:00 | 33.81 | 16.42 | 11.83 | 0.03 | 239.70 | 12271.29 | 31.84 |
| a-3-13.8-0:30 | 22.05 | 9.70 | 13.56 | 0.05 | 368.01 | 17075.36 | 20.08 |
| a-3-13.8-1:30 | 21.97 | 9.67 | 15.20 | 0.05 | 436.48 | 19653.60 | 20.00 |
| a-3-13.8-2:30 | 26.38 | 12.08 | 17.97 | 0.05 | 497.22 | 23420.27 | 24.41 |
| b-1-2.9-0:00 | 44.28 | 22.24 | 16.83 | 0.03 | 326.93 | 20572.31 | 42.31 |
| b-1-2.9-0:30 | 35.80 | 16.48 | 29.31 | 0.07 | 1046.85 | 36207.56 | 33.83 |
| b-1-2.9-1:30 | 26.23 | 10.80 | 34.84 | 0.11 | 1388.43 | 43265.86 | 24.26 |
| b-1-2.9-2:30 | 25.93 | 9.96 | 40.30 | 0.13 | 1748.03 | 50139.66 | 23.96 |
| b-2-11-0:00 | 194.60 | 112.25 | 116.76 | 0.16 | 4468.98 | 43466.94 | 192.63 |
| b-2-11-0:30 | 205.03 | 122.86 | 105.19 | 0.13 | 3660.45 | 40501.41 | 203.06 |
| b-2-11-1:30 | 221.51 | 140.75 | 83.15 | 0.10 | 2378.54 | 32237.17 | 219.54 |
| b-2-11-2:30 | 246.70 | 172.58 | 61.60 | 0.07 | 1368.60 | 23964.67 | 244.73 |
| b-3-13.8-0:00 | 40.61 | 21.14 | 17.05 | 0.03 | 350.70 | 20049.07 | 38.64 |
| b-3-13.8-0:30 | 26.41 | 12.09 | 20.69 | 0.06 | 614.06 | 26675.66 | 24.44 |
| b-3-13.8-1:30 | 26.35 | 12.06 | 14.15 | 0.04 | 347.22 | 18563.41 | 24.38 |
| b-3-13.8-2:30 | 26.35 | 12.06 | 1.17 | 0.00 | 8.22 | 1533.53 | 24.38 |
