# Supplementary material for: Applicability of Different Hydraulic Parameters to Describe Soil Detachment in Eroding Rills
Source: PLoS One. 2013 May 24;8(5):e64861. doi: 10.1371/journal.pone.0064861 (PMC3663750; doi:10.1371/journal.pone.0064861)
Supplement: Table S10 — Negratin erosion data. (DOC) [file pone.0064861.s010.doc]

Table S10 Negratin erosion data

| Run - MP - flow length [m]- sampling time [min:sec] | Sediment Concentration [g L^-1^] | Detachment rate [kg s^-1^ m^-2^] | Transport rate [kg s^-1^] | Sample density [g cm^-3^] | Slope [°] | Transport capacity [kg s^-1^] |
| --- | --- | --- | --- | --- | --- | --- |
| a-1-3.2-0:00 | 67.3 | 0.3509 | 0.378972891 | 1.04 | 3.2 | 0.02284 |
| a-1-3.2-0:30 | 55.7 | 0.3594 | 0.417851939 | 1.03 | 3.2 | 0.02926 |
| a-1-3.2-1:30 | 37.0 | 0.2853 | 0.331751054 | 1.02 | 3.2 | 0.02877 |
| a-1-3.2-2:30 | 35.2 | 0.3353 | 0.411477439 | 1.02 | 3.2 | 0.03395 |
| a-2-5.1-0:00 | 128.8 | 0.1191 | 0.083361100 | 1.08 | 7.7 | 0.00238 |
| a-2-5.1-0:30 | 102.6 | 0.2553 | 0.258820005 | 1.06 | 7.7 | 0.01888 |
| a-2-5.1-1:30 | 60.6 | 0.1840 | 0.183346450 | 1.04 | 7.7 | 0.01584 |
| a-2-5.1-2:30 | 41.3 | 0.1941 | 0.219213489 | 1.03 | 7.7 | 0.02768 |
| a-3-11.5-0:00 | 170.4 | 0.0370 | 0.039566213 | 1.11 | 1.7 | 0.00012 |
| a-3-11.5-0:30 | 155.3 | 0.0391 | 0.039947665 | 1.10 | 1.7 | 0.00009 |
| a-3-11.5-1:30 | 95.0 | 0.0333 | 0.035583905 | 1.06 | 1.7 | 0.00011 |
| a-3-11.5-2:30 | 81.0 | 0.0364 | 0.040214682 | 1.05 | 1.7 | 0.00016 |
| b-1-3.2-0:00 | 82.1 | 0.5253 | 0.567410676 | 1.05 | 3.2 | 0.02314 |
| b-1-3.2-0:30 | 35.9 | 0.2925 | 0.340134613 | 1.02 | 3.2 | 0.02874 |
| b-1-3.2-1:30 | 24.7 | 0.2555 | 0.297103809 | 1.02 | 3.2 | 0.02845 |
| b-1-3.2-2:30 | 12.4 | 0.2164 | 0.265524812 | 1.01 | 3.2 | 0.03324 |
| b-2-5.1-0:00 | 128.2 | 0.1551 | 0.112506225 | 1.08 | 7.7 | 0.00302 |
| b-2-5.1-0:30 | 48.3 | 0.1641 | 0.171502072 | 1.03 | 7.7 | 0.01942 |
| b-2-5.1-1:30 | 28.8 | 0.1376 | 0.147576853 | 1.02 | 7.7 | 0.02288 |
| b-2-5.1-2:30 | 28.3 | 0.1823 | 0.219256736 | 1.02 | 7.7 | 0.03540 |
| b-3-11.5-0:00 | 168.5 | 0.0358 | 0.038265903 | 1.10 | 1.7 | 0.00012 |
| b-3-11.5-0:30 | 79.8 | 0.0234 | 0.025837099 | 1.05 | 1.7 | 0.00016 |
| b-3-11.5-1:30 | 52.8 | 0.0137 | 0.013972868 | 1.03 | 1.7 | 0.00008 |
| b-3-11.5-2:30 | 51.4 | 0.0153 | 0.016371852 | 1.03 | 1.7 | 0.00011 |
