# Supplementary material for: Applicability of Different Hydraulic Parameters to Describe Soil Detachment in Eroding Rills
Source: PLoS One. 2013 May 24;8(5):e64861. doi: 10.1371/journal.pone.0064861 (PMC3663750; doi:10.1371/journal.pone.0064861)
Supplement: Table S11 — Negratin runoff data. (DOC) [file pone.0064861.s011.doc]

Table S11 Negratin runoff data

| Run - MP - flow length [m]- sampling time [min:sec] | Flow velocity [m s^-1^] | Dynamic viscosity [kg s^-1^ m^-1^] | Water depth [cm] | Flow cross section [cm²] | Wetted Perimeter [cm] | Hydraulic radius [cm] |
| --- | --- | --- | --- | --- | --- | --- |
| a-1-3.2-0:00 | 0.53 | 0.001336 | 0.5 | 106.28 | 33.75 | 3.15 |
| a-1-3.2-0:30 | 0.59 | 0.001278 | 2 | 126.85 | 36.33 | 3.49 |
| a-1-3.2-1:30 | 0.71 | 0.001185 | 2 | 126.85 | 36.33 | 3.49 |
| a-1-3.2-2:30 | 0.82 | 0.001176 | 3 | 143.13 | 38.35 | 3.73 |
| a-2-5.1-0:00 | 0.64 | 0.001644 | 0.4 | 10.11 | 13.72 | 0.74 |
| a-2-5.1-0:30 | 0.75 | 0.001513 | 2.1 | 33.86 | 19.88 | 1.70 |
| a-2-5.1-1:30 | 0.96 | 0.001303 | 2 | 31.49 | 19.53 | 1.61 |
| a-2-5.1-2:30 | 1.18 | 0.001207 | 2.6 | 44.94 | 22.14 | 2.03 |
| a-3-11.5-0:00 | 0.46 | 0.001852 | 0.40 | 5.05 | 9.30 | 0.54 |
| a-3-11.5-0:30 | 0.59 | 0.001776 | 0.30 | 4.33 | 8.89 | 0.49 |
| a-3-11.5-1:30 | 0.74 | 0.001475 | 0.40 | 5.05 | 9.30 | 0.54 |
| a-3-11.5-2:30 | 0.81 | 0.001405 | 0.50 | 6.12 | 9.61 | 0.64 |
| b-1-3.2-0:00 | 0.65 | 0.001411 | 0.5 | 106.28 | 33.75 | 3.15 |
| b-1-3.2-0:30 | 0.75 | 0.001180 | 2 | 126.85 | 36.33 | 3.49 |
| b-1-3.2-1:30 | 0.95 | 0.001123 | 2 | 126.85 | 36.33 | 3.49 |
| b-1-3.2-2:30 | 1.50 | 0.001062 | 3 | 143.13 | 38.35 | 3.73 |
| b-2-5.1-0:00 | 0.76 | 0.001641 | 0.5 | 11.54 | 14.22 | 0.81 |
| b-2-5.1-0:30 | 0.99 | 0.001242 | 2.3 | 36.00 | 20.49 | 1.76 |
| b-2-5.1-1:30 | 1.29 | 0.001144 | 2.5 | 39.73 | 21.03 | 1.89 |
| b-2-5.1-2:30 | 1.46 | 0.001141 | 3 | 53.06 | 23.58 | 2.25 |
| b-3-11.5-0:00 | 0.45 | 0.001843 | 0.40 | 5.05 | 9.30 | 0.54 |
| b-3-11.5-0:30 | 0.53 | 0.001399 | 0.50 | 6.12 | 9.61 | 0.64 |
| b-3-11.5-1:30 | 0.61 | 0.001264 | 0.30 | 4.33 | 8.89 | 0.49 |
| b-3-11.5-2:30 | 0.63 | 0.001257 | 0.40 | 5.05 | 9.30 | 0.54 |
