# Supplementary material for: Applicability of Different Hydraulic Parameters to Describe Soil Detachment in Eroding Rills
Source: PLoS One. 2013 May 24;8(5):e64861. doi: 10.1371/journal.pone.0064861 (PMC3663750; doi:10.1371/journal.pone.0064861)
Supplement: Table S12 — Negratin hydraulic data. (DOC) [file pone.0064861.s012.doc]

Table S12 Negratin hydraulic data

| Run - MP - flow length [m]- sampling time [min:sec] | τ [Pa] | Г [N m^-1^] | ω [W m^-2^] | ω_U_ [m s^-1^] | ω_eff_ [W m^-1^] | Re [ ] | τ - τ_cr_ [Pa] |
| --- | --- | --- | --- | --- | --- | --- | --- |
| a-1-3.2-0:00 | 17.96 | 6.06 | 9.52 | 0.03 | 1004.67 | 13009.48 | 15.04 |
| a-1-3.2-0:30 | 19.78 | 7.19 | 11.70 | 0.03 | 543.29 | 16715.29 | 16.85 |
| a-1-3.2-1:30 | 19.56 | 7.11 | 13.84 | 0.04 | 698.89 | 21333.97 | 16.63 |
| a-1-3.2-2:30 | 20.89 | 8.01 | 17.08 | 0.05 | 730.82 | 26518.86 | 17.96 |
| a-2-5.1-0:00 | 10.46 | 1.44 | 6.70 | 0.09 | 687.70 | 3099.18 | 7.54 |
| a-2-5.1-0:30 | 23.82 | 4.74 | 17.75 | 0.10 | 982.41 | 8925.34 | 20.90 |
| a-2-5.1-1:30 | 21.99 | 4.30 | 21.12 | 0.13 | 1317.11 | 12329.08 | 19.06 |
| a-2-5.1-2:30 | 27.36 | 6.06 | 32.28 | 0.16 | 2090.11 | 20354.33 | 24.44 |
| a-3-11.5-0:00 | 1.75 | 0.16 | 0.80 | 0.01 | 28.58 | 1490.41 | -1.18 |
| a-3-11.5-0:30 | 1.56 | 0.14 | 0.92 | 0.02 | 42.67 | 1786.38 | -1.37 |
| a-3-11.5-1:30 | 1.67 | 0.16 | 1.24 | 0.02 | 54.86 | 2890.54 | -1.26 |
| a-3-11.5-2:30 | 1.95 | 0.19 | 1.58 | 0.02 | 67.92 | 3863.99 | -0.98 |
| b-1-3.2-0:00 | 18.12 | 6.12 | 11.78 | 0.04 | 1382.74 | 15249.04 | 15.20 |
| b-1-3.2-0:30 | 19.55 | 7.10 | 14.58 | 0.04 | 755.90 | 22575.90 | 16.62 |
| b-1-3.2-1:30 | 19.41 | 7.05 | 18.44 | 0.05 | 1074.74 | 29978.88 | 16.48 |
| b-1-3.2-2:30 | 20.60 | 7.90 | 30.90 | 0.08 | 1778.67 | 53133.31 | 17.67 |
| b-2-5.1-0:00 | 11.52 | 1.64 | 8.76 | 0.10 | 886.05 | 4058.91 | 8.59 |
| b-2-5.1-0:30 | 23.79 | 4.87 | 23.45 | 0.13 | 1404.17 | 14369.29 | 20.86 |
| b-2-5.1-1:30 | 25.28 | 5.32 | 32.62 | 0.17 | 2178.77 | 21693.14 | 22.35 |
| b-2-5.1-2:30 | 30.10 | 7.10 | 44.00 | 0.20 | 3022.79 | 29329.14 | 27.17 |
| b-3-11.5-0:00 | 1.74 | 0.16 | 0.79 | 0.01 | 27.61 | 1464.08 | -1.18 |
| b-3-11.5-0:30 | 1.94 | 0.19 | 1.03 | 0.02 | 35.72 | 2528.39 | -0.98 |
| b-3-11.5-1:30 | 1.47 | 0.13 | 0.90 | 0.02 | 40.73 | 2434.20 | -1.46 |
| b-3-11.5-2:30 | 1.63 | 0.15 | 1.03 | 0.02 | 41.37 | 2810.22 | -1.30 |
