# Supplementary material for: Applicability of Different Hydraulic Parameters to Describe Soil Detachment in Eroding Rills
Source: PLoS One. 2013 May 24;8(5):e64861. doi: 10.1371/journal.pone.0064861 (PMC3663750; doi:10.1371/journal.pone.0064861)
Supplement: Table S13 — Salada erosion data. (DOC) [file pone.0064861.s013.doc]

Table S13 Salada erosion data

| Run - MP - flow length [m]- sampling time [min:sec] | Sediment Concentration [g L^-1^] | Detachment rate [kg s^-1^ m^-2^] | Transport rate [kg s^-1^] | Sample density [g cm^-3^] | Slope [°] | Transport capacity [kg s^-1^] |
| --- | --- | --- | --- | --- | --- | --- |
| a-1-2.3-0:00 | 65.7 | 0.2744 | 0.163638470 | 1.04 | 14.9 | 0.07299 |
| a-1-2.3-0:30 | 21.9 | 0.0840 | 0.053634448 | 1.01 | 14.9 | 0.12230 |
| a-1-2.3-1:30 | 11.4 | 0.0767 | 0.044566797 | 1.01 | 14.9 | 0.05316 |
| a-1-2.3-2:30 | 15.4 | 0.4565 | 0.309609576 | 1.01 | 14.9 | 0.18004 |
| a-2-4.7-0:00 | 30.8 | 0.0812 | 0.094584461 | 1.02 | 24.5 | 0.14384 |
| a-2-4.7-0:30 | 18.3 | 0.0529 | 0.070550578 | 1.01 | 24.5 | 0.29789 |
| a-2-4.7-1:30 | 40.7 | 0.2666 | 0.437682105 | 1.03 | 24.5 | 0.79787 |
| a-2-4.7-2:30 | 11.4 | 0.0980 | 0.117807544 | 1.01 | 24.5 | 0.17785 |
| a-3-4.7-0:00 | 113.4 | 0.3238 | 0.315564275 | 1.07 | 24.5 | 0.18940 |
| a-3-4.7-0:30 | 42.2 | 0.1193 | 0.126580737 | 1.03 | 24.5 | 0.28476 |
| a-3-4.7-1:30 | 25.0 | 0.1295 | 0.149733833 | 1.02 | 24.5 | 0.43935 |
| a-3-4.7-2:30 | 14.7 | 0.2187 | 0.282982246 | 1.01 | 24.5 | 0.70410 |
| b-1-2.3-0:00 | 36.8 | 0.1280 | 0.076308115 | 1.02 | 14.9 | 0.07110 |
| b-1-2.3-0:30 | 8.1 | 0.0812 | 0.091376454 | 1.01 | 14.9 | 0.25308 |
| b-1-2.3-1:30 | 5.8 | 0.0783 | 0.081788592 | 1.00 | 14.9 | 0.23377 |
| b-1-2.3-2:30 | 7.0 | 0.1136 | 0.143162068 | 1.00 | 14.9 | 0.36293 |
| b-2-4.7-0:00 | 42.9 | 0.2433 | 0.404671007 | 1.03 | 24.5 | 0.84780 |
| b-2-4.7-0:30 | 19.7 | 0.1253 | 0.189952323 | 1.01 | 24.5 | 0.54642 |
| b-2-4.7-1:30 | 9.6 | 0.0684 | 0.084120916 | 1.01 | 24.5 | 0.20340 |
| b-2-4.7-2:30 | 10.9 | 0.1511 | 0.215740177 | 1.01 | 24.5 | 0.41807 |
| b-3-4.7-0:00 | 39.0 | 0.1429 | 0.151710680 | 1.02 | 24.5 | 0.28391 |
| b-3-4.7-0:30 | 9.5 | 0.0551 | 0.063738761 | 1.01 | 24.5 | 0.43310 |
| b-3-4.7-1:30 | 7.8 | 0.0905 | 0.117090916 | 1.00 | 24.5 | 0.69957 |
| b-3-4.7-2:30 | 6.6 | 0.1257 | 0.174190550 | 1.00 | 24.5 | 0.90918 |
