# Supplementary material for: Applicability of Different Hydraulic Parameters to Describe Soil Detachment in Eroding Rills
Source: PLoS One. 2013 May 24;8(5):e64861. doi: 10.1371/journal.pone.0064861 (PMC3663750; doi:10.1371/journal.pone.0064861)
Supplement: Table S14 — Salada runoff data. (DOC) [file pone.0064861.s014.doc]

Table S14 Salada runoff data

| Run - MP - flow length [m]- sampling time [min:sec] | Flow velocity [m s^-1^] | Dynamic viscosity [kg s^-1^ m^-1^] | Water depth [cm] | Flow cross section [cm²] | Wetted Perimeter [cm] | Hydraulic radius [cm] |
| --- | --- | --- | --- | --- | --- | --- |
| a-1-2.3-0:00 | 0.48 | 0.001328 | 2.5 | 51.91 | 25.92 | 2.00 |
| a-1-2.3-0:30 | 0.35 | 0.001110 | 3.2 | 69.41 | 27.75 | 2.50 |
| a-1-2.3-1:30 | 0.86 | 0.001057 | 2.2 | 45.47 | 25.27 | 1.80 |
| a-1-2.3-2:30 | 2.33 | 0.001077 | 4.1 | 86.29 | 29.49 | 2.93 |
| a-2-4.7-0:00 | 0.62 | 0.001154 | 4.7 | 49.50 | 24.79 | 2.00 |
| a-2-4.7-0:30 | 0.51 | 0.001091 | 3.7 | 76.10 | 28.35 | 2.68 |
| a-2-4.7-1:30 | 0.78 | 0.001204 | 7.7 | 137.92 | 34.93 | 3.95 |
| a-2-4.7-2:30 | 1.84 | 0.001057 | 4.1 | 56.01 | 25.58 | 2.19 |
| a-3-4.7-0:00 | 0.62 | 0.001567 | 5 | 44.87 | 20.73 | 2.16 |
| a-3-4.7-0:30 | 0.51 | 0.001211 | 6 | 59.01 | 22.58 | 2.61 |
| a-3-4.7-1:30 | 0.78 | 0.001125 | 7 | 76.97 | 24.61 | 3.13 |
| a-3-4.7-2:30 | 1.84 | 0.001073 | 8 | 104.38 | 27.53 | 3.79 |
| b-1-2.3-0:00 | 0.40 | 0.001184 | 2.5 | 51.91 | 25.92 | 2.00 |
| b-1-2.3-0:30 | 0.68 | 0.001041 | 6.3 | 164.46 | 48.91 | 3.36 |
| b-1-2.3-1:30 | 0.95 | 0.001029 | 5.9 | 148.03 | 45.41 | 3.26 |
| b-1-2.3-2:30 | 0.96 | 0.001035 | 7.5 | 212.97 | 54.81 | 3.89 |
| b-2-4.7-0:00 | 0.66 | 0.001214 | 8.0 | 143.01 | 35.38 | 4.04 |
| b-2-4.7-0:30 | 0.87 | 0.001098 | 6.6 | 110.25 | 32.24 | 3.42 |
| b-2-4.7-1:30 | 1.45 | 0.001048 | 4.2 | 60.45 | 26.15 | 2.31 |
| b-2-4.7-2:30 | 2.12 | 0.001054 | 5.7 | 93.66 | 30.39 | 3.08 |
| b-3-4.7-0:00 | 0.66 | 0.001195 | 6 | 59.01 | 22.58 | 2.61 |
| b-3-4.7-0:30 | 0.87 | 0.001047 | 7 | 76.97 | 24.61 | 3.13 |
| b-3-4.7-1:30 | 1.45 | 0.001039 | 8 | 104.38 | 27.53 | 3.79 |
| b-3-4.7-2:30 | 2.12 | 0.001033 | 9 | 124.17 | 29.48 | 4.21 |
