# Supplementary material for: Applicability of Different Hydraulic Parameters to Describe Soil Detachment in Eroding Rills
Source: PLoS One. 2013 May 24;8(5):e64861. doi: 10.1371/journal.pone.0064861 (PMC3663750; doi:10.1371/journal.pone.0064861)
Supplement: Table S15 — Salada hydraulic data. (DOC) [file pone.0064861.s015.doc]

Table S15 Salada hydraulic data

| Run - MP - flow length [m]- sampling time [min:sec] | τ [Pa] | Г [N m^-1^] | ω [W m^-2^] | ω_U_ [m s^-1^] | ω_eff_ [W m^-1^] | Re [ ] | τ - τ_cr_ [Pa] |
| --- | --- | --- | --- | --- | --- | --- | --- |
| a-1-2.3-0:00 | 52.57 | 13.63 | 25.24 | 0.12 | 1482.75 | 7531.25 | 49.37 |
| a-1-2.3-0:30 | 63.95 | 17.75 | 22.53 | 0.09 | 1061.25 | 8050.56 | 60.75 |
| a-1-2.3-1:30 | 45.71 | 11.55 | 39.35 | 0.22 | 3144.11 | 14760.25 | 42.50 |
| a-1-2.3-2:30 | 74.52 | 21.98 | 173.86 | 0.60 | 19280.91 | 64004.64 | 71.32 |
| a-2-4.7-0:00 | 82.79 | 20.52 | 51.33 | 0.26 | 2823.76 | 10932.84 | 79.59 |
| a-2-4.7-0:30 | 110.44 | 31.31 | 56.09 | 0.21 | 3783.55 | 12635.30 | 107.23 |
| a-2-4.7-1:30 | 164.68 | 57.53 | 128.40 | 0.32 | 8038.43 | 26224.38 | 161.48 |
| a-2-4.7-2:30 | 89.70 | 22.95 | 165.43 | 0.76 | 17895.79 | 38472.08 | 86.50 |
| a-3-4.7-0:00 | 94.27 | 19.54 | 58.44 | 0.26 | 3292.10 | 9166.87 | 91.06 |
| a-3-4.7-0:30 | 109.10 | 24.64 | 55.42 | 0.21 | 2691.66 | 11247.11 | 105.90 |
| a-3-4.7-1:30 | 129.22 | 31.80 | 100.75 | 0.32 | 5953.66 | 22018.10 | 126.02 |
| a-3-4.7-2:30 | 155.65 | 42.85 | 287.09 | 0.76 | 26199.37 | 65737.82 | 152.45 |
| b-1-2.3-0:00 | 51.66 | 13.39 | 20.67 | 0.10 | 1098.76 | 6920.70 | 48.46 |
| b-1-2.3-0:30 | 85.25 | 41.70 | 58.17 | 0.18 | 2802.12 | 22158.58 | 82.04 |
| b-1-2.3-1:30 | 82.54 | 37.48 | 78.04 | 0.24 | 4548.92 | 30059.69 | 79.33 |
| b-1-2.3-2:30 | 98.44 | 53.96 | 94.08 | 0.25 | 5131.14 | 36030.09 | 95.24 |
| b-2-4.7-0:00 | 168.82 | 59.74 | 111.42 | 0.27 | 6334.72 | 22554.07 | 165.62 |
| b-2-4.7-0:30 | 140.82 | 45.40 | 123.21 | 0.36 | 8374.20 | 27572.20 | 137.61 |
| b-2-4.7-1:30 | 94.60 | 24.74 | 136.77 | 0.60 | 13237.98 | 32076.24 | 91.40 |
| b-2-4.7-2:30 | 126.24 | 38.36 | 267.99 | 0.88 | 29621.25 | 62486.08 | 123.04 |
| b-3-4.7-0:00 | 108.88 | 24.59 | 71.86 | 0.27 | 3974.97 | 14785.43 | 105.68 |
| b-3-4.7-0:30 | 127.99 | 31.50 | 111.99 | 0.36 | 6977.44 | 26284.46 | 124.79 |
| b-3-4.7-1:30 | 154.99 | 42.67 | 224.07 | 0.60 | 18065.59 | 53022.45 | 151.78 |
| b-3-4.7-2:30 | 172.07 | 50.72 | 365.28 | 0.88 | 34762.59 | 86918.88 | 168.87 |
