# Supplementary material for: Applicability of Different Hydraulic Parameters to Describe Soil Detachment in Eroding Rills
Source: PLoS One. 2013 May 24;8(5):e64861. doi: 10.1371/journal.pone.0064861 (PMC3663750; doi:10.1371/journal.pone.0064861)
Supplement: Table S16 — Belerda erosion data. (DOC) [file pone.0064861.s016.doc]

Table S16 Belerda erosion data

| Run - MP - flow length [m]- sampling time [min:sec] | Sediment Concentration [g L^-1^] | Detachment rate [kg s^-1^ m^-2^] | Transport rate [kg s^-1^] | Sample density [g cm^-3^] | Slope [°] | Transport capacity [kg s^-1^] |
| --- | --- | --- | --- | --- | --- | --- |
| a-1-6-0:00 | 111.1 | 0.6584 | 0.463968090 | 1.07 | 11.7 | 0.01464 |
| a-1-6-0:30 | 250.8 | 0.9576 | 0.674872393 | 1.15 | 11.7 | 0.01644 |
| a-1-6-1:30 | 288.4 | 0.5173 | 0.364531283 | 1.18 | 11.7 | 0.01694 |
| a-1-6-2:30 | 176.1 | 0.4415 | 0.387089673 | 1.11 | 11.7 | 0.04693 |
| a-2-13-0:00 | 243.8 | 0.1124 | 0.242154919 | 1.15 | 15.1 | 0.03603 |
| a-2-13-0:30 | 359.6 | 0.1026 | 0.084365950 | 1.22 | 15.1 | 0.00926 |
| a-2-13-1:30 | 234.8 | 0.0617 | 0.038866537 | 1.14 | 15.1 | 0.00230 |
| a-2-13-2:30 | 88.0 | 0.0533 | 0.041378347 | 1.05 | 15.1 | 0.00577 |
| a-3-17-0:00 | 341.2 | 0.2734 | 0.876936459 | 1.21 | 15.4 | 0.15169 |
| a-3-17-0:30 | 422.3 | 0.6314 | 2.058803483 | 1.26 | 15.4 | 0.16862 |
| a-3-17-1:30 | 62.6 | 0.0887 | 0.297213079 | 1.04 | 15.4 | 0.13513 |
| a-3-17-2:30 | 383.4 | 0.0896 | 0.296150064 | 1.24 | 15.4 | 0.16984 |
| b-1-6-0:00 | 93.0 | 0.1522 | 0.118800584 | 1.06 | 11.7 | 0.02513 |
| b-1-6-0:30 | 212.6 | 0.3207 | 0.226026124 | 1.13 | 11.7 | 0.01594 |
| b-1-6-1:30 | 16.5 | 0.0211 | 0.012073263 | 1.01 | 11.7 | 0.00496 |
| b-1-6-2:30 | 13.5 | 0.0389 | 0.030357874 | 1.01 | 11.7 | 0.02340 |
| b-2-13-0:00 | 312.2 | 0.2399 | 0.516754731 | 1.19 | 15.1 | 0.03803 |
| b-2-13-0:30 | 97.3 | 0.0387 | 0.038785872 | 1.06 | 15.1 | 0.01574 |
| b-2-13-1:30 | 421.1 | 0.2325 | 0.171978951 | 1.26 | 15.1 | 0.00611 |
| b-2-13-2:30 | 61.8 | 0.0860 | 0.086292771 | 1.04 | 15.1 | 0.01525 |
| b-3-17-0:00 | 31.7 | 0.0240 | 0.078397448 | 1.02 | 15.4 | 0.12267 |
| b-3-17-0:30 | 141.3 | 0.1565 | 0.524266143 | 1.09 | 15.4 | 0.14471 |
| b-3-17-1:30 | 125.3 | 0.2185 | 0.722405305 | 1.08 | 15.4 | 0.13812 |
| b-3-17-2:30 | 109.2 | 0.2682 | 0.909694568 | 1.07 | 15.4 | 0.14510 |
