# Supplementary material for: Applicability of Different Hydraulic Parameters to Describe Soil Detachment in Eroding Rills
Source: PLoS One. 2013 May 24;8(5):e64861. doi: 10.1371/journal.pone.0064861 (PMC3663750; doi:10.1371/journal.pone.0064861)
Supplement: Table S17 — Belerda runoff data. (DOC) [file pone.0064861.s017.doc]

Table S17 Belerda runoff data

| Run - MP - flow length [m]- sampling time [min:sec] | Flow velocity [m s^-1^] | Dynamic viscosity [kg s^-1^ m^-1^] | Water depth [cm] | Flow cross section [cm²] | Wetted Perimeter [cm] | Hydraulic radius [cm] |
| --- | --- | --- | --- | --- | --- | --- |
| a-1-6-0:00 | 2.94 | 0.001556 | 1.0 | 14.20 | 11.75 | 1.21 |
| a-1-6-0:30 | 1.89 | 0.002254 | 1.0 | 14.20 | 11.75 | 1.21 |
| a-1-6-1:30 | 0.89 | 0.002442 | 1.0 | 14.20 | 11.75 | 1.21 |
| a-1-6-2:30 | 0.80 | 0.001881 | 2.0 | 27.55 | 14.61 | 1.89 |
| a-2-13-0:00 | 0.42 | 0.002219 | 3.0 | 23.65 | 16.57 | 1.43 |
| a-2-13-0:30 | 0.46 | 0.002798 | 0.7 | 5.06 | 6.33 | 0.80 |
| a-2-13-1:30 | 0.72 | 0.002174 | 0.2 | 2.31 | 4.85 | 0.48 |
| a-2-13-2:30 | 1.09 | 0.001440 | 0.6 | 4.32 | 5.98 | 0.72 |
| a-3-17-0:00 | 0.56 | 0.002706 | 0.2 | 45.89 | 18.87 | 2.43 |
| a-3-17-0:30 | 1.03 | 0.003111 | 0.3 | 47.50 | 19.18 | 2.48 |
| a-3-17-1:30 | 0.95 | 0.001313 | 0.5 | 50.15 | 19.70 | 2.55 |
| a-3-17-2:30 | 0.16 | 0.002917 | 0.4 | 48.85 | 19.45 | 2.51 |
| b-1-6-0:00 | 0.65 | 0.001465 | 1.5 | 19.65 | 13.01 | 1.51 |
| b-1-6-0:30 | 0.75 | 0.002063 | 1.0 | 14.20 | 11.75 | 1.21 |
| b-1-6-1:30 | 0.94 | 0.001083 | 0.2 | 7.72 | 9.52 | 0.81 |
| b-1-6-2:30 | 1.14 | 0.001068 | 1.5 | 19.65 | 13.01 | 1.51 |
| b-2-13-0:00 | 0.70 | 0.002561 | 3.0 | 23.65 | 16.57 | 1.43 |
| b-2-13-0:30 | 0.48 | 0.001486 | 1.0 | 8.31 | 7.72 | 1.08 |
| b-2-13-1:30 | 1.08 | 0.003105 | 0.5 | 3.78 | 5.69 | 0.66 |
| b-2-13-2:30 | 1.68 | 0.001309 | 1.0 | 8.31 | 7.72 | 1.08 |
| b-3-17-0:00 | 0.52 | 0.001159 | 0.3 | 47.50 | 19.18 | 2.48 |
| b-3-17-0:30 | 0.74 | 0.001706 | 0.5 | 50.15 | 19.70 | 2.55 |
| b-3-17-1:30 | 1.18 | 0.001627 | 0.4 | 48.85 | 19.45 | 2.51 |
| b-3-17-2:30 | 1.62 | 0.001546 | 0.6 | 51.41 | 19.95 | 2.58 |
