# Supplementary material for: Applicability of Different Hydraulic Parameters to Describe Soil Detachment in Eroding Rills
Source: PLoS One. 2013 May 24;8(5):e64861. doi: 10.1371/journal.pone.0064861 (PMC3663750; doi:10.1371/journal.pone.0064861)
Supplement: Table S18 — Belerda hydraulic data. (DOC) [file pone.0064861.s018.doc]

Table S18 Belerda hydraulic data

| Run - MP - flow length [m]- sampling time [min:sec] | τ [Pa] | Г [N m^-1^] | ω [W m^-2^] | ω_U_ [m s^-1^] | ω_eff_ [W m^-1^] | Re [ ] | τ - τ_cr_ [Pa] |
| --- | --- | --- | --- | --- | --- | --- | --- |
| a-1-6-0:00 | 25.71 | 3.02 | 75.58 | 0.60 | 14155.26 | 24423.50 | 22.94 |
| a-1-6-0:30 | 27.78 | 3.26 | 52.62 | 0.38 | 8224.56 | 11735.83 | 25.01 |
| a-1-6-1:30 | 28.34 | 3.33 | 25.22 | 0.18 | 2728.19 | 5190.96 | 25.57 |
| a-1-6-2:30 | 41.58 | 6.08 | 33.17 | 0.16 | 2592.39 | 8864.96 | 38.81 |
| a-2-13-0:00 | 41.96 | 6.95 | 17.62 | 0.11 | 766.31 | 3107.72 | 39.19 |
| a-2-13-0:30 | 24.97 | 1.58 | 11.58 | 0.12 | 1076.71 | 1619.26 | 22.19 |
| a-2-13-1:30 | 13.93 | 0.67 | 10.00 | 0.19 | 1991.25 | 1799.58 | 11.16 |
| a-2-13-2:30 | 19.47 | 1.16 | 21.19 | 0.28 | 2954.62 | 5758.07 | 16.70 |
| a-3-17-0:00 | 76.70 | 14.47 | 42.95 | 0.15 | 17731.56 | 6092.17 | 73.92 |
| a-3-17-0:30 | 81.32 | 15.60 | 83.46 | 0.27 | 36654.75 | 10296.35 | 78.55 |
| a-3-17-1:30 | 68.88 | 13.57 | 65.17 | 0.25 | 17991.78 | 19049.17 | 66.11 |
| a-3-17-2:30 | 80.93 | 15.74 | 12.80 | 0.04 | 1816.39 | 1683.83 | 78.16 |
| b-1-6-0:00 | 31.78 | 4.13 | 20.66 | 0.13 | 1543.50 | 7087.10 | 29.01 |
| b-1-6-0:30 | 27.21 | 3.20 | 20.36 | 0.15 | 1979.84 | 4961.43 | 24.44 |
| b-1-6-1:30 | 16.31 | 1.55 | 15.41 | 0.19 | 3811.70 | 7155.59 | 13.54 |
| b-1-6-2:30 | 30.30 | 3.94 | 34.59 | 0.23 | 3345.39 | 16287.61 | 27.53 |
| b-2-13-0:00 | 43.50 | 7.21 | 30.45 | 0.18 | 1740.33 | 4652.71 | 40.73 |
| b-2-13-0:30 | 29.16 | 2.25 | 14.00 | 0.13 | 1128.08 | 3684.88 | 26.39 |
| b-2-13-1:30 | 21.39 | 1.22 | 23.11 | 0.28 | 3798.42 | 2911.66 | 18.62 |
| b-2-13-2:30 | 28.56 | 2.20 | 47.98 | 0.44 | 7159.25 | 14340.51 | 25.79 |
| b-3-17-0:00 | 65.77 | 12.62 | 34.20 | 0.14 | 9616.37 | 11330.97 | 63.00 |
| b-3-17-0:30 | 72.10 | 14.20 | 53.35 | 0.20 | 13326.36 | 12001.08 | 69.32 |
| b-3-17-1:30 | 70.51 | 13.71 | 83.19 | 0.31 | 30113.84 | 19632.98 | 67.74 |
| b-3-17-2:30 | 71.65 | 14.29 | 116.06 | 0.43 | 37864.55 | 28811.68 | 68.87 |
