# Supplementary material for: Applicability of Different Hydraulic Parameters to Describe Soil Detachment in Eroding Rills
Source: PLoS One. 2013 May 24;8(5):e64861. doi: 10.1371/journal.pone.0064861 (PMC3663750; doi:10.1371/journal.pone.0064861)
Supplement: Table S19 — R2 - values between the detachment rate and different hydraulic parameters. (DOC) [file pone.0064861.s019.doc]

Table S19 R² - values between the detachment rate and different hydraulic parameters. MP = measuring point, τ = shear stress, Г = unit length shear force, ω = stream power, ω_U_ = unit stream power, ω_eff_ = effective stream power, Re = Reynolds number, τ_cr_ = critical shear stress, + indicates increasing trend line, - indicates decreasing trend line, 0 indicates a nearly constant trend line.

|  |  | **τ** |  | **Г** |  | **ω** |  | **ω_U_** |  | **ω_eff_** |  | **Re** |  | **τ - τ_cr_** |  |
| --- | --- | --- | --- | --- | --- | --- | --- | --- | --- | --- | --- | --- | --- | --- | --- |
| Freila 1 run a | MP 1 | 0.29 | 0 | 0.12 | 0 | 0.02 | - | 0.02 | - | 0.00 | 0 | 0.02 | - | 0.29 | 0 |
|  | MP 2 | 0.49 | 0 | 0.55 | 0 | 0.49 | + | 0.01 | 0 | 0.48 | + | 0.49 | + | 0.49 | 0 |
|  | MP 3 | 0.18 | + | 0.18 | + | 0.01 | + | 0.94 | - | 0.01 | - | 0.02 | - | 0.18 | + |
| Freila 1 run b | MP 1 | 0.03 | 0 | 0.06 | 0 | 0.41 | - | 0.47 | - | 0.33 | - | 0.51 | - | 0.03 | 0 |
|  | MP 2 | 0.95 | 0 | 0.98 | 0 | 0.95 | + | 0.30 | 0 | 0.94 | + | 0.95 | + | 0.95 | 0 |
|  | MP 3 | 0.16 | + | 0.15 | + | 0.00 | 0 | 0.15 | - | 0.02 | - | 0.00 | 0 | 0.16 | + |
| Freila 2 run a | MP 1 | 0.81 | + | 0.91 | + | 0.17 | + | 0.45 | - | 0.32 | - | 0.01 | + | 0.81 | + |
|  | MP 2 | 0.08 | - | 0.08 | - | 0.58 | - | 0.61 | - | 0.37 | - | 0.61 | - | 0.08 | 0 |
|  | MP 3 | 0.8 | - | 0.79 | - | 0.97 | - | 0.78 | - | 0.94 | - | 0.98 | - | 0.80 | - |
| Freila 2 run b | MP 1 | 0.84 | - | 0.85 | - | 0.99 | - | 0.84 | - | 0.92 | - | 0.99 | - | 0.84 | - |
|  | MP 2 | 0.99 | - | 0.99 | - | 0.58 | - | 0.53 | - | 0.19 | - | 0.62 | - | 0.99 | - |
|  | MP 3 | 0.92 | - | 0.92 | - | 0.22 | - | 0.08 | - | 0.13 | - | 0.27 | - | 0.92 | - |
| Freila 3 run a | MP 1 | 0.97 | + | 0.98 | + | 0.14 | - | 0.69 | - | 0.43 | - | 0.36 | - | 0.97 | - |
|  | MP 2 | 0.88 | - | 0.82 | - | 0.89 | - | 0.92 | + | 0.89 | - | 0.94 | - | 0.88 | - |
|  | MP 3 | 0.80 | + | 0.81 | + | 0.62 | - | 0.99 | - | 0.86 | - | 0.78 | - | 0.80 | + |
| Freila 3 run b | MP 1 | 0.96 | + | 0.96 | + | 0.93 | - | 0.99 | - | 0.94 | - | 0.94 | - | 0.96 | + |
|  | MP 2 | 0.64 | - | 0.60 | - | 0.68 | + | 0.72 | + | 0.74 | + | 0.79 | + | 0.64 | - |
|  | MP 3 | 0.93 | + | 0.93 | + | 0.26 | + | 0.03 | + | 0.09 | + | 0.19 | + | 0.93 | + |
| Negratin run a | MP 1 | 0.03 | - | 0.03 | - | 0.16 | - | 0.23 | - | 0.01 | + | 0.20 | - | 0.03 | - |
|  | MP 2 | 0.60 | + | 0.53 | + | 0.22 | + | 0.05 | + | 0.06 | + | 0.14 | + | 0.60 | + |
|  | MP 3 | 0.31 | - | 0.09 | + | 0.19 | + | 0.96 | + | 0.15 | 0 | 0.23 | 0 | 0.05 | 0 |
| Negratin run b | MP 1 | 0.86 | - | 0.86 | - | 0.53 | - | 0.51 | - | 0.00 | 0 | 0.58 | - | 0.86 | - |
|  | MP 2 | 0.14 | + | 0.19 | + | 0.16 | + | 0.08 | + | 0.17 | 0 | 0.14 | + | 0.14 | + |
|  | MP 3 | 0.31 | + | 0.27 | + | 0.39 | + | 0.24 | + | 0.99 | 0 | 0.79 | 0 | 0.31 | 0 |
| Salada run a | MP 1 | 0.44 | + | 0.45 | + | 0.70 | + | 0.64 | + | 0.70 | + | 0.67 | + | 0.44 | + |
|  | MP 2 | 0.78 | + | 0.81 | + | 0.22 | + | 0.00 | 0 | 0.02 | + | 0.12 | + | 0.78 | + |
|  | MP 3 | 0.09 | - | 0.07 | - | 0.00 | 0 | 0.02 | 0 | 0.01 | 0 | 0.00 | 0 | 0.09 | - |
| Salada run b | MP 1 | 0.23 | - | 0.18 | - | 0.19 | - | 0.27 | - | 0.17 | - | 0.20 | - | 0.23 | - |
|  | MP 2 | 0.86 | + | 0.87 | + | 0.01 | - | 0.18 | - | 0.05 | - | 0.03 | - | 0.86 | + |
|  | MP 3 | 0.00 | - | 0.00 | - | 0.03 | 0 | 0.02 | 0 | 0.05 | 0 | 0.02 | 0 | 0.00 | 0 |
| Belerda run a | MP 1 | 0.35 | - | 0.35 | - | 0.25 | + | 0.28 | + | 0.26 | + | 0.08 | + | 0.34 | - |
|  | MP 2 | 0.71 | + | 0.55 | + | 0.02 | 0 | 0.85 | - | 0.92 | - | 0.23 | - | 0.71 | + |
|  | MP 3 | 0.27 | + | 0.19 | + | 0.47 | + | 0.31 | + | 0.78 | + | 0.00 | 0 | 0.27 | + |
| Belerda run b | MP 1 | 0.12 | + | 0.07 | + | 0.05 | - | 0.46 | - | 0.59 | - | 0.37 | - | 0.12 | + |
|  | MP 2 | 0.06 | + | 0.20 | + | 0.00 | 0 | 0.01 | 0 | 0.02 | - | 0.16 | - | 0.06 | + |
|  | MP 3 | 0.71 | + | 0.74 | + | 0.88 | + | 0.86 | + | 0.83 | + | 0.72 | + | 0.71 | + |
