# Supplementary material for: Applicability of Different Hydraulic Parameters to Describe Soil Detachment in Eroding Rills
Source: PLoS One. 2013 May 24;8(5):e64861. doi: 10.1371/journal.pone.0064861 (PMC3663750; doi:10.1371/journal.pone.0064861)
Supplement: Table S20 — Comparison of the transport rate with the transport capacity: Transport rate TR [kg s−1] - Transport capacity TC [kg s−1]. (DOC) [file pone.0064861.s020.doc]

Table S20 Comparison of the transport rate with the transport capacity: Transport rate T_R_ [kg s^-1^] - Transport capacity T_C_ [kg s^-1^]

| run-MP-time | Freila1 | Freila 2 | Freila 3 | Negratin | Salada | Belerda |
| --- | --- | --- | --- | --- | --- | --- |
| a - 1 - 0:00 | 0.016496 | 0.208367 | 0.299736 | 0.356133 | 0.090648 | 0.449332 |
| a - 1 - 0:30 | 0.009216 | -0.000042 | -0.002630 | 0.388589 | -0.068663 | 0.658430 |
| a - 1 - 1:30 | 0.028119 | -0.009571 | -0.011526 | 0.302982 | -0.008596 | 0.347591 |
| a - 1 - 2:30 | 0.003356 | 0.008419 | -0.018163 | 0.377529 | 0.129568 | 0.340157 |
| a - 2 - 0:00 | 0.000700 | 0.016861 | -0.122188 | 0.080985 | -0.049255 | 0.206128 |
| a - 2 - 0:30 | 0.000594 | -0.003886 | -1.661911 | 0.239944 | -0.227338 | 0.075105 |
| a - 2 - 1:30 | 0.000078 | -0.003910 | -2.256325 | 0.167504 | -0.360192 | 0.036567 |
| a - 2 - 2:30 | -0.070511 | -0.009096 | -3.036197 | 0.191535 | -0.060043 | 0.035613 |
| a - 3 - 0:00 | 0.218935 | 0.034645 | 0.337938 | 0.039447 | 0.126165 | 0.725246 |
| a - 3 - 0:30 | 0.036412 | -0.594298 | 0.059926 | 0.039858 | -0.158184 | 1.890184 |
| a - 3 - 1:30 | 0.012344 | -0.618707 | 0.021373 | 0.035472 | -0.289620 | 0.162081 |
| a - 3 - 2:30 | 0.005022 | -0.637078 | 0.000277 | 0.040050 | -0.421114 | 0.126305 |
| b - 1 - 0:00 | 0.058696 | 0.033665 | -0.109527 | 0.544266 | 0.005208 | 0.093669 |
| b - 1 - 0:30 | -0.000152 | 0.000836 | -0.062758 | 0.311392 | -0.161699 | 0.210084 |
| b - 1 - 1:30 | 0.000136 | -0.005232 | -0.033282 | 0.268657 | -0.151983 | 0.007109 |
| b - 1 - 2:30 | -0.001141 | -0.007456 | -0.038459 | 0.232281 | -0.219765 | 0.006954 |
| b - 2 - 0:00 | 0.000120 | 0.027868 | -1.587432 | 0.109482 | -0.443133 | 0.478729 |
| b - 2 - 0:30 | -0.000007 | -0.011142 | -2.043042 | 0.152084 | -0.356465 | 0.023048 |
| b - 2 - 1:30 | -0.068115 | -0.014524 | -2.539236 | 0.124697 | -0.119275 | 0.165872 |
| b - 2 - 2:30 | -0.001477 | -0.013833 | -3.356041 | 0.183860 | -0.202332 | 0.071039 |
| b - 3 - 0:00 | 0.001240 | 0.188280 | 0.178267 | 0.038147 | -0.132203 | -0.044270 |
| b - 3 - 0:30 | 0.003910 | -0.641102 | 0.033766 | 0.025673 | -0.369358 | 0.379557 |
| b - 3 - 1:30 | -0.198913 | -0.656871 | -0.020963 | 0.013890 | -0.582478 | 0.584281 |
| b - 3 - 2:30 | -0.230061 | -0.693729 | -0.041146 | 0.016264 | -0.734992 | 0.764599 |
