# Supplementary material for: Applicability of Different Hydraulic Parameters to Describe Soil Detachment in Eroding Rills
Source: PLoS One. 2013 May 24;8(5):e64861. doi: 10.1371/journal.pone.0064861 (PMC3663750; doi:10.1371/journal.pone.0064861)
Supplement: Table S21 — Comparison of the transport rate with the transport capacity: Percentage of TR exceeding TC. (DOC) [file pone.0064861.s021.doc]

Table S21 Comparison of the transport rate with the transport capacity: Percentage of T_R_ exceeding T_C_

| run-MP-time | Freila1 | Freila 2 | Freila 3 | Negratin | Salada | Belerda |
| --- | --- | --- | --- | --- | --- | --- |
| a - 1 - 0:00 | 92.9 | 78.0 | 78.9 | 94.0 | 55.4 | 96.8 |
| a - 1 - 0:30 | 87.8 | 0 | 0 | 93.0 | 0 | 97.6 |
| a - 1 - 1:30 | 95.6 | 0 | 0 | 91.3 | 0 | 95.4 |
| a - 1 - 2:30 | 72.7 | 42.6 | 0 | 91.7 | 41.8 | 87.9 |
| a - 2 - 0:00 | 96.5 | 49.9 | 0 | 97.1 | 0 | 85.1 |
| a - 2 - 0:30 | 65.0 | 0 | 0 | 92.7 | 0 | 89.0 |
| a - 2 - 1:30 | 4.8 | 0 | 0 | 91.4 | 0 | 94.1 |
| a - 2 - 2:30 | 0 | 0 | 0 | 87.4 | 0 | 86.1 |
| a - 3 - 0:00 | 91.1 | 12.0 | 81.4 | 99.7 | 40.0 | 82.7 |
| a - 3 - 0:30 | 86.8 | 0 | 68.8 | 99.8 | 0 | 91.8 |
| a - 3 - 1:30 | 43.5 | 0 | 44.1 | 99.7 | 0 | 54.5 |
| a - 3 - 2:30 | 20.4 | 0 | 0.6 | 99.6 | 0 | 42.6 |
| b - 1 - 0:00 | 97.8 | 90.3 | 0 | 95.9 | 6.8 | 78.8 |
| b - 1 - 0:30 | 0 | 5.8 | 0 | 91.5 | 0 | 92.9 |
| b - 1 - 1:30 | 9.7 | 0 | 0 | 90.4 | 0 | 58.9 |
| b - 1 - 2:30 | 0 | 0 | 0 | 87.5 | 0 | 22.9 |
| b - 2 - 0:00 | 94.9 | 62.1 | 0 | 97.3 | 0 | 92.6 |
| b - 2 - 0:30 | 0 | 0 | 0 | 88.7 | 0 | 59.4 |
| b - 2 - 1:30 | 0 | 0 | 0 | 84.5 | 0 | 96.4 |
| b - 2 - 2:30 | 0 | 0 | 0 | 83.9 | 0 | 82.3 |
| b - 3 - 0:00 | 0.5 | 42.2 | 59.0 | 99.7 | 0 | 0 |
| b - 3 - 0:30 | 32.5 | 0 | 44.1 | 99.4 | 0 | 72.4 |
| b - 3 - 1:30 | 0 | 0 | 0 | 99.4 | 0 | 80.9 |
| b - 3 - 2:30 | 0 | 0 | 0 | 99.3 | 0 | 84.1 |
